# Ablation of an Ovarian Tumor Family Deubiquitinase Exposes the Underlying Regulation Governing the Plasticity of Cell Cycle Progression in *Toxoplasma gondii*

**DOI:** 10.1128/mBio.01846-17

**Published:** 2017-11-21

**Authors:** Animesh Dhara, Rodrigo de Paula Baptista, Jessica C. Kissinger, E. Charles Snow, Anthony P. Sinai

**Affiliations:** a Department of Microbiology, Immunology and Molecular Genetics, University of Kentucky College of Medicine, Lexington, Kentucky, USA; b Center for Tropical and Emerging Global Diseases, University of Georgia, Athens, Georgia, USA; c Institute of Bioinformatics, University of Georgia, Athens, Georgia, USA; d Department of Genetics, University of Georgia, Athens, Georgia, USA; Albert Einstein College of Medicine

**Keywords:** deubiquitinase, *Toxoplasma gondii*, apicomplexan parasites, cell cycle, centrosomes

## Abstract

The *Toxoplasma* genome encodes the capacity for distinct architectures underlying cell cycle progression in a life cycle stage-dependent manner. Replication in intermediate hosts occurs by endodyogeny, whereas a hybrid of schizogony and endopolygeny occurs in the gut of the definitive feline host. Here, we characterize the consequence of the loss of a cell cycle-regulated ovarian tumor (OTU family) deubiquitinase, OTUD3A of *Toxoplasma gondii* (TgOTUD3A; TGGT1_258780), in *T. gondii* tachyzoites. Rather than the mutation being detrimental, mutant parasites exhibited a fitness advantage, outcompeting the wild type. This phenotype was due to roughly one-third of TgOTUD3A-knockout (TgOTUD3A-KO) tachyzoites exhibiting deviations from endodyogeny by employing replication strategies that produced 3, 4, or 5 viable progeny within a gravid mother instead of the usual 2. We established the mechanistic basis underlying these altered replication strategies to be a dysregulation of centrosome duplication, causing a transient loss of stoichiometry between the inner and outer cores that resulted in a failure to terminate S phase at the attainment of 2N ploidy and/or the decoupling of mitosis and cytokinesis. The resulting dysregulation manifested as deviations in the normal transitions from S phase to mitosis (S/M) (endopolygeny-like) or M phase to cytokinesis (M/C) (schizogony-like). Notably, these imbalances are corrected prior to cytokinesis, resulting in the generation of normal progeny. Our findings suggest that decisions regarding the utilization of specific cell cycle architectures are controlled by a ubiquitin-mediated mechanism that is dependent on the absolute threshold levels of an as-yet-unknown target(s). Analysis of the TgOTUD3A-KO mutant provides new insights into mechanisms underlying the plasticity of apicomplexan cell cycle architecture.

## IMPORTANCE

Replication by *Toxoplasma gondii* can occur by 3 distinct cell cycle architectures. Endodyogeny is used by asexual stages, while a hybrid of schizogony and endopolygeny is used by merozoites in the definitive feline host. Here, we establish that the disruption of an ovarian-tumor (OTU) family deubiquitinase, TgOTUD3A, in tachyzoites results in dysregulation of the mechanism controlling the selection of replication strategy in a subset of parasites. The mechanistic basis for these altered cell cycles lies in the unique biology of the bipartite centrosome that is associated with the transient loss of stoichiometry between the inner and outer centrosome cores in the TgOTUD3A-KO mutant. This highlights the importance of ubiquitin-mediated regulation in the transition from the nuclear to the budding phases of the cell cycle and provides new mechanistic insights into the regulation of the organization of the apicomplexan cell cycle.

## INTRODUCTION

Parasites of the phylum Apicomplexa are responsible for numerous diseases of both human and veterinary importance (1, 2). At the heart of their pathogenesis is their ability to replicate intracellularly within their hosts. Apicomplexa exhibit diverse replication strategies that are defined by the organization of their cell cycles, which are more complex than those of higher eukaryotes (3). The apicomplexan cell cycle is defined by two distinct phases: the nuclear cycle (comprising DNA synthesis, mitosis, and karyokinesis) and the budding cycle (comprising cytokinesis) (see Fig. S1 in the supplemental material). This architecture accounts for the capacity of these parasites to uncouple the nuclear cycle from the budding cycle depending on the replication strategy employed (3).

10.1128/mBio.01846-17.2 FIG S1  Apicomplexan replication strategies. Endodyogeny. Organization is linear and unidirectional with overlapping cell cycle phase (G_1_ = Gap1, S = DNA synthesis, M = mitosis, and C = cytokinesis). The nuclear and the budding cycles are tightly integrated and produce 2 daughters at the end of each cycle (for example, *Toxoplasma*). Schizogony. Organization consists of several rounds of asynchronous mitosis and karyokinesis resulting from the completion of the nuclear cycle without entry into the budding cycle. The final round of synchronous nuclear cycle is coupled with the budding cycle, resulting in the formation of an even number of multiple progeny (for example, *Plasmodium*). Endopolygeny. Organization consists of multiple rounds of DNA replication and mitosis (segregation of genome equivalents within the nuclear mass) in the absence of karyokinesis to generate a polyploid nuclear mass. The final round of DNA synthesis results in large-scale mitosis coupled to karyokinesis that is linked to cytokinesis. This represents entry into the budding cycle and the formation of daughter parasites. In the case of *Sarcocystis neurona*, 32 rounds of the incomplete nuclear cycle and in the last round an overlapping nuclear and budding cycle produce a total of 64 progeny. Download FIG S1, TIF file, 0.2 MB. Copyright © 2017 Dhara et al. 2017 Dhara et al. This content is distributed under the terms of the Creative Commons Attribution 4.0 International license.

*Toxoplasma gondii*, an important member of the Apicomplexa, is of particular concern in immunocompromised individuals and as a source of congenitally acquired infections (4). *Toxoplasma* parasites exhibit the capacity to alter their replication strategy, with tachyzoites (associated with the acute asexual phase) (5) and bradyzoites (associated with the chronic asexual phase) replicating by endodyogeny (6, 7), while the replication of merozoites in the feline intestine occurs by a hybrid of schizogony and endopolygeny (8, 9). As such, the *Toxoplasma* genome encodes the capacity to replicate by all 3 major apicomplexan cell cycle mechanisms.

Endodyogeny is an internal budding mechanism by which 2 daughters are formed within the mother (Fig. S1) (5, 7). In the course of endodyogeny, each cycle is associated with a single round of DNA synthesis, progressing to mitosis and karyokinesis, which overlaps with cytokinesis, resulting in the formation of the daughter parasites within the mother cell (3, 10). In contrast, *Plasmodium* spp., the agents of malaria, replicate by schizogony wherein rounds of DNA synthesis and mitosis/karyokinesis in the absence of cytokinesis result in a multinucleated cell that then undergoes synchronous cytokinesis (budding cycle), resulting in an even number of progeny (Fig. S1) (11). The third apicomplexan strategy is endopolygeny, (e.g., *Sarcocystis* spp.), in which the nuclear cycle is defined by successive rounds of DNA synthesis and what appears to be mitosis (segregation of replicated genomes) without the execution of karyokinesis (nuclear division), resulting in the formation of a polyploid nuclear mass within the corpus of the parasite (3). Upon entry into the budding cycle (cytokinesis), the execution of karyokinesis results in the packaging of individual nuclei into the forming daughter scaffolds, which allows the birth of multiple progeny (64 in the case of *Sarcocystis neurona*) (Fig. S1) (3). The mechanistic basis underlying the capacity of *Toxoplasma* to employ each of these three replication strategies in a life cycle stage-dependent context has not been explored.

The replication of eukaryotes is a highly regulated unidirectional process that is defined by the cell cycle (12, 13). Tight control is achieved by the spatiotemporal regulation of protein complexes governed by phosphorylation/dephosphorylation (14, 15) and protein stabilization/turnover (15). The latter mechanism is orchestrated predominantly by a balance between ubiquitination and deubiquitination of specific targets (16), driven by a complex interplay between ubiquitin ligases and deubiquitinases (DUBs) (17, 18).

The importance of ubiquitin-mediated mechanisms in the regulation of the *Toxoplasma* cell cycle can be inferred by the disproportionate number of cell cycle-regulated genes whose products are subject to ubiquitination (19). Ubiquitination itself is a highly versatile posttranslational modification based on the covalent linkage of ubiquitin (Ub) via one of 7 possible lysine residues (K6, K11, K27, K29, K33, K48, and K63) to a target protein that defines the ubiquitin code (20, 21). The resultant combinatorial diversity observed in polyUb structures provides a mechanism to achieve a very high degree of functional specificity (22) that is catalyzed by the activity of specific Ub ligases (23). On the other side of the equation, the deubiquitinases (DUBs) present a similar range of specificities regarding the Ub chains that they selectively target (18). This permits an exquisite level of fine control that not only governs the stability/turnover of the target protein but can also have nondegradative signaling and trafficking functions (24–26). The contribution of DUBs in the fine-tuning of the cell cycle in higher eukaryotes is well documented (27–29). How the ubiquitin cycle regulates the diverse replication strategies of Apicomplexa remains unexplored.

Here, we focus on the role ubiquitination plays as a means for controlling the fidelity of *Toxoplasma* tachyzoite replication. While several studies have looked into the contribution of ubiquitin ligases in Apicomplexa (30–32), investigations on deubiquitinases are limited (30). We reasoned that DUBs that exhibit a strong cell cycle-dependent transcriptional expression profile are more likely to be involved in the regulation of cell cycle progression. One gene, encoding a member of the ovarian tumor (OTU) family of DUBs (33), which we designated TgOTUD3A (*T. gondii* OTUD3A), exhibits tight cell cycle-dependent gene and protein expression (34). The functional characterization of recombinant TgOTUD3A confirms its enzymatic activity and specificity (for K48-linked polyUb) (34). Somewhat to our surprise, the genetic ablation of TgOTUD3A failed to generate a gross growth defect despite a clear defect within a subset of parasites in the fidelity of endodyogeny. In these instances, the generation of multiple daughter parasites often exhibited typical features of schizogony and endopolygeny, suggestive of a partial shift in cell cycle architecture.

Characterization of the TgOTUD3A-knockout (TgOTUD3A-KO) mutant exposes an important role for a ubiquitin-mediated process in regulating the selection of the replication strategy. Here, we investigate how the mechanistic basis underlying the selection of the replication strategy is connected to alterations in the dynamics of the bipartite *Toxoplasma* centrosome (35), particularly as it relates to the control of the transition from the nuclear to the budding phase of the cell cycle. This work establishes a framework for a better understanding of the complexity inherent in the architecture of the apicomplexan cell cycle.

## RESULTS

### *T. gondii* with ablation of TgOTUD3A does not exhibit a gross growth defect.

**FIG 1:**
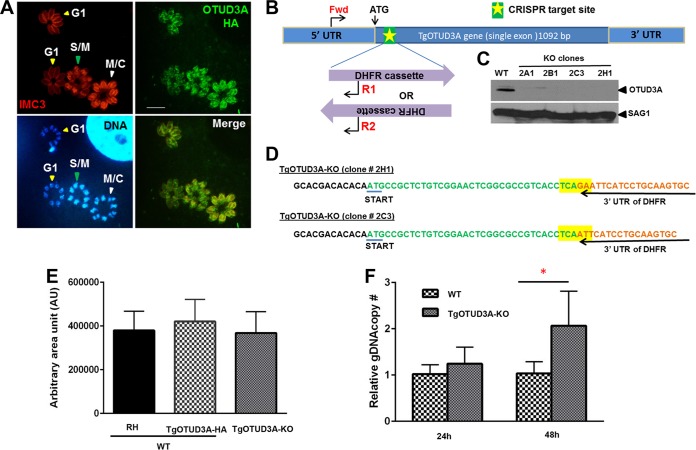
Cell cycle dynamics of *T. gondii* TgOTUD3A wild type and growth phenotype of TgOTUD3A-KO mutant. (A) Distribution and relative levels of TgOTUD3A-HA as functions of cell cycle progression, visualized using TgIMC3, nuclear morphology, and labeling intensity. TgOTUD3A-HA levels increase with the progression of the cell cycle from G_1_ through S/M, peaking at the end of mitosis and with the progression of cytokinesis. TgOTUD3A-HA localizes to the cytoplasm and associates with daughter scaffolds when present. The large labeled structure in the top right corner of the DNA panel is the nucleus of the infected host cell. Scale bars = 10 µm. (B) Location of the site targeted for CRISPR-Cas9-mediated cleavage at the TgOTUD3A genomic locus just downstream from the ATG start codon. The TgOTUD3A-targeting CRISPR–Cas9-GFP construct was cotransfected with a DNA sequence encoding the DHFR cassette, which can integrate into the CRISPR break site in two different orientations. Integration of DHFR into the locus was confirmed using the indicated PCR primers (red, Fwd/R1 and Fwd/R2). (C) Immunoblot analysis of four pyrimethamine-resistant clones (2A1, 2B1, 2C3, and 2H1) with polyclonal TgOTUD3A mouse antibody reveals a loss of TgOTUD3A protein expression to confirm the TgOTUD3A knockout. Independent clones 2C3 and 2H1 were characterized in this study. Protein from wild-type (WT) parasites was run as a positive antibody control. (D) Sequence analysis of theTgOTUD3A-KO DHFR insertion junction confirms that 2C3 and 2H1 are independent clones. Sequence differences are highlighted in yellow. (E) Effect of TgOTUD3A knockout on growth assessed using a plaque assay: the areas of approximately 150 randomly acquired plaques (~50 plaques for 3 different lines each from three independent experiments) generated at 6 days postinfection by wild-type (either the RH or TgOTUD3A-HA-tagged line) and TgOTUD3A-KO parasites were measured and plotted. The mean plaque sizes of wild-type (RH and TgOTUD3A-HA) and TgOTUD3A-KO parasites were not statistically significant by one-way ANOVA (F_2,150_ = 4.094, *P* < 0.0186). (F) A head-to-head competition assay (see Materials and Methods for details) was performed. Data compiled from 4 independent experiments confirm that there were mean 1.2-fold and 2-fold increases in the DNA content of TgOTUD3A-KO parasites relative to the DNA content of the wild type (TgOTUD3-HA) at 24 h and 48 h postinfection. When analyzed with ANOVA (F_3,12_ = 5.085, *P* < 0.0168) and multiple comparisons, the average gDNA copy number increase at 48 h was found to be significant, suggesting some fitness advantage of the TgOTUD3A-KO mutant over the wild type. *, *P* ≤ 0.01. Error bars represent standard deviations.

The progression of the *Toxoplasma gondii* tachyzoite cell cycle can be morphologically tracked based on the appearance of the nucleus and the development of the daughter scaffolds, detected using inner membrane complex (IMC) proteins as markers within the mother parasite undergoing endodyogeny. Parasites in G_1_ possessed a compact nucleus and weak labeling of the maternal inner membrane complex when visualized using the *T. gondii* IMC3 (TgIMC3) marker (Fig. 1A). Entry and progression into S phase was characterized by increased size and intensity of the parasite nuclei, which toward the end of S phase and the onset of mitosis (S/M) possessed a bilobed, heart-shaped configuration (Fig. 1A, DNA). In light of the overlapping organization of the stages in endodyogeny (10), the early daughter buds were present during the initial phases of mitosis and are seen as the bright TgIMC3-labeled structures (Fig. 1A, IMC3, red). The completion of mitosis was evident from the resolution of the bilobed nuclear mass into two discrete nuclei and the expansion of the daughter buds into mature scaffolds, eventually resulting in the emergence of two new parasites upon the recycling of maternal components (Fig. 1A). TgOTUD3A is a cell cycle-regulated deubiquitinase of the ovarian tumor family (33), the levels of which increased upon entry into S phase, reaching peak levels during cytokinesis, when they appeared to be associated with the developing daughters (Fig. 1A) (34). This pattern of protein expression mirrored that of its gene transcription (36), suggesting a potentially important function in cell cycle progression. We used a clustered regularly interspaced short palindromic repeat–CRISPR-associated protein 9 (CRISPR-Cas9) system that has been adapted to *Toxoplasma gondii* (37) to introduce a knock-in mutation into the open reading frame of the TgOTUD3A gene, just downstream from the ATG start codon, using a dihydrofolate reductase (DHFR) cassette that confers pyrimethamine resistance (Pyr^r^) (Fig. 1B). We were able to select multiple knockout clones that were confirmed using immunoblot analysis (Fig. 1C), with the insertion and orientation of the drug resistance cassette confirmed by PCR (data not shown), using primers Fwd/R1 and Fwd/R2 (Fig. 1B), and sequencing of the amplicons (Fig. 1D). The recovery of viable TgOTUD3A-KO clones indicated that the gene was nonessential. Further sequencing of the clones confirmed the integration of DHFR, and sequence variation at the integration sites confirmed that 2H1 and 2C3 were independent clonal lines (Fig. 1D). We sought to establish whether the loss of TgOTUD3A had any detrimental effect on parasite infectivity and growth using a plaque assay. Surprisingly, despite its being tightly cell cycle regulated (34) (Fig. 1A), mutants with the ablation of TgOTUD3A had no apparent growth defects relative to the growth of the wild-type (WT) parental (RH) or the hemagglutinin (HA)-tagged line (in an RHΔKu80 background [34]) (Fig. 1E). We next conducted a head-to-head competition assay (see Materials and Methods for details), in which the TgOTUD3A-KO mutants consistently outcompeted the wild-type line, with mean increases of 1.2- and 2-fold, respectively, at 24 and 48 h postinfection (Fig. 1F). Of note, this fitness advantage was reported for TgOTUD3A-KO in a recently published genome-wide CRISPR-based competition screen for essentiality (38).

This unexpected result led us to perform cell cycle analysis based on DNA content for both wild-type and mutant parasites using flow cytometry. Although small shifts in the proportions of parasites exhibiting >2N ploidy were observed for the TgOTUD3A-KO parasites, their levels were not statistically significant (Fig. S2). This indicated that increased ploidy alone was not the sole driver of the higher genome equivalents observed in the mutant. We therefore assessed whether the observed increase in fitness was due to an actual increase in parasite numbers.

10.1128/mBio.01846-17.3 FIG S2  DNA content analysis by FACS. DNA content analysis of the wild-type and TgOTUD3A-KO parasites, established using flow cytometry of syringe-passaged and washed parasites labeled with the DNA binding dye SYTOX green. The DNA content (ploidy) of actively growing wild-type and TgOTUD3A-KO parasites was established 24 and 36 h postinfection. The parasite signal was established based on the forward and side scatter characteristics for a comparison of uninfected syringe-passaged cells to infected cells (not shown). Relative SYTOX intensity (*y* axis) was used to define the DNA content (ploidy), and counting gates were established for 1N (G_1_), 1N to 2N (S phase), 2N (premitotic), and >2N (polyploid) levels of DNA. Polyploidy within a parasite can be due to its possessing a single >2N nucleus or multiple individual nuclei, the total DNA content of which is >2N. The distribution in the dot plots was analyzed using WinList 8.0 software and presented as a histogram. The relative percentage of parasites in each gate is presented in the table adjacent to the histogram. No statistically significant difference in the overall ploidy of the population distribution was observed between wild-type and TgOTUD3A-KO parasites at the time points tested. The data are from a representative experiment of the 3 replicates that were performed. Download FIG S2, TIF file, 0.4 MB. Copyright © 2017 Dhara et al. 2017 Dhara et al. This content is distributed under the terms of the Creative Commons Attribution 4.0 International license.

### Increased asynchrony and abnormal endodyogeny in a subset of TgOTUD3A-KO mutants are associated with dysregulation of the nuclear cycle.

**FIG 2:**
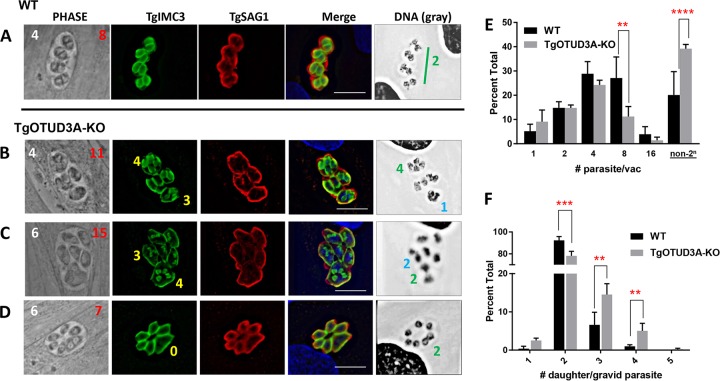
TgOTUD3A-KO parasites exhibit a range of altered/aberrant endodyogeny phenotypes. WT and TgOTUD3A-KO parasites in HFFs were fixed at 24 h postinfection and labeled with rat anti-TgIMC3 and rabbit anti-TgSAG1 antibodies. Nuclear DNA was labeled with Hoechst dye. The DNA images were converted into gray scale for better visualization. Phase images show parasite morphology, with total numbers of parasites (white numbers) and of mature/immature daughter scaffolds (red numbers) per vacuole indicated. (A) Wild-type parasites exhibit synchronous 2^*n*^ replication as determined by the number of daughter scaffolds (TgIMC3, green) in each replicating parasite (TgSAG1, red), representing normal endodyogeny. (B and C) The replication phenotypes in TgOTUD3A-KO parasites are often altered with regard to the total number (non-2^*n*^) of parasites within vacuoles, the numbers of daughter scaffolds within individual mothers, the numbers of nuclei, and the nuclear morphology. In each case presented where daughter scaffolds are present, aberrant and normal endodyogeny are evident within the same vacuole. In the DNA images, symmetrical nuclear division is indicated with green numbers, while polyploid and asymmetrical nuclei within individual parasites are marked with blue numbers. In the Phase and TgSAG1 images, note that mother parasites containing 3 or 4 TgIMC3-positive (yellow numbers in TgIMC3 images) daughter scaffolds tend to be enlarged and morphologically distinct relative to mother parasites that are undergoing normal endodyogeny. In the case of multiple daughters, all the daughter scaffolds within a given mother were found to be of equal size. (D) Evidence of mitosis (nuclei marked with green 2 in DNA image) in the absence of linked cytokinesis suggests a disconnect between nuclear and budding cycles. (A to D) Scale bars = 10 µm. (E) Quantification of aberrant replication. Parasitophorous vacuoles (PV) were labeled with the PV marker mouse anti-TgGRA3 antibody, while individual parasites were identified using the surface marker rabbit anti-TgSAG1 antibody. All slides were coded, and the numbers of parasites per vacuole (2^*n*^ [1, 2, 4, 8, and 16] versus non-2^*n*^) were counted blindly by three individuals. A total of 4 biological replicates with 3 independent samples per replicate were counted. One-way ANOVA indicated that there was a significant difference (F_11,36_ = P < 0.0001) between the wild-type and TgOTUD3A-KO lines with regard to the incidence of deviation from a 2^*n*^ progression that manifests following the first 2 rounds of replication. A total of 1,649 vacuoles (WT = 756 and KO = 893) were counted for this analysis. (F) Comparison between numbers of daughter scaffolds (TgIMC3, green) per gravid parasite (TgSAG1, red) counted blindly in both wild-type and TgOTUD3A-KO parasites reveals that a significantly higher proportion of gravid mother parasites bear >2 progeny in the TgOTUD3A-KO than in the parental (WT) line. Analysis was restricted to parasites that contained daughter scaffolds (TgIMC3 positive). One-way ANOVA indicated significant differences (F_9,25_ = 761.8, *P* < 0.0001) between wild-type and KO parasites. (E and F) Tukey’s pairwise multiple-comparison test (α = 0.05) was used to compare the level of significance for comparison between wild-type and KO parasites for each group analyzed. Significant differences (*P* ≤ 0.05) are indicated by asterisks as follows: **, *P* ≤ 0.01; ***, *P* ≤ 0.001; ****, *P* ≤ 0.0001. A total of 2,059 (WT = 980, KO = 1,079) gravid parasites were counted in this analysis. Error bars represent standard deviations of the means.

A feature of tachyzoite replication is the geometric increase in parasite numbers due to a high degree of synchrony, resulting in their following a 2^*n*^ progression for parasite growth within individual vacuoles. This outcome was seen in the example of the wild type, where four mother parasites (Fig. 2A, Phase, white number 4) each contained two forming daughters, resulting in eight progeny within the vacuole (Fig. 2A, Phase, red number 8). This normal geometric progression, however, was differentially altered in a subset of TgOTUD3A-KO parasites that exhibited non-2^*n*^ numbers of parasites per vacuole (Fig. 2C and D, Phase, white numbers).

Deviation from a 2^*n*^ progression of parasites per vacuole could be explained either by the loss of replicative synchrony or the production of more than two daughters per mother parasite per replicative cycle. Such deviation from normal endodyogeny has been reported to occur at a low level in wild-type parasites and to be enhanced under specific conditions (5, 39, 40), and it also occurs in conditional mutants with mutations affecting the cell cycle (41). We therefore examined the number of daughter scaffolds within gravid mother parasites within vacuoles using TgIMC3. As opposed to the wild-type parasites, where each gravid mother typically contains two daughters, each with an equally divided nucleus (Fig. 2A), a subset of the TgOTUD3A-KO parasites exhibited a diverse range of phenotypes, often within the same vacuoles, as presented in Fig. 2B to D. Examination of the daughter scaffold burden (TgIMC3) revealed the presence of mothers with two (not marked), three, and four daughter scaffolds, respectively. In many instances, the mother parasites containing two scaffolds appeared to have progressed further along cytokinesis than those with three and four daughter scaffolds, based on the size of the TgIMC3 scaffolds (Fig. 2B). In other vacuoles, particularly those containing relatively immature scaffolds, the sizes of forming daughters with 2, 3, and 4 scaffolds were comparable (Fig. 2B and C). What was uniform, however, was that the size of daughter scaffolds within a given parasite (whether 2, 3, or 4) was always the same, suggesting coordination of the initiation and progression of cytokinesis within a given mother (Fig. 2B and C). In addition, despite the differences in the numbers of daughters within individual members of a vacuole, all of the parasites within a vacuole were gravid, as partial gravidity was rarely observed. Thus, despite individual parasites within a vacuole displaying differing numbers of developing daughters, they appeared to be synchronized with regard to transitioning from the nuclear to the budding cycle (cytokinesis).

Insights into the patterns of replication were also provided from the nuclear morphology, best observed in the grayscale rendition of the DNA (Hoechst-stained) images. For mother parasites containing 3 scaffolds (Fig. 2B and C), 3 distinct nuclear states were evident. The first contained a single large polyploid nucleus that was denser than the other nuclei within the same vacuole (Fig. 2B, DNA, blue number 1). The second state displayed 2 asymmetrical nuclei within the mother, one small and one large, that differed in both size and density (Fig. 2C, DNA, blue number 2). Finally, a state where the 3 daughter scaffolds were accompanied by 3 equivalently sized postmitotic nuclei (Fig. S3A) suggested the initial dysregulation was/could be corrected to produce multiple progeny from the gravid mother. For mother parasites containing 4 daughter scaffolds, 2 distinct nuclear morphologies were evident. The first possessed 2 equivalently sized larger nuclei, with the small immature daughter buds suggesting a pending second round of nuclear division (karyokinesis) (Fig. 2C, DNA, green number 2). The second nuclear morphology exhibited 4 distinct nuclei suggestive of a postkaryokinesis state and associated with well-developed daughter TgIMC3 scaffolds (Fig. 2B, DNA, green number 4).

10.1128/mBio.01846-17.4 FIG S3  Additional examples of aberrant endodyogeny phenotypes associated with the TgOTUD3A-KO line. Replication phenotypes of TgOTUD3A-KO parasites were examined. The number of daughter scaffolds (TgIMC3, green) and nuclear morphology (DNA Hoechst dye) in each replicating parasite (TgSAG1, red) undergoing replication were monitored. The main replication phenotypes are presented in Fig. 2. Some additional examples are presented here. (A, B) TgOTUD3A-KO parasites exhibiting aberrant endodyogeny and multiple-daughter phenotypes within the same vacuole where normal endodyogeny is progressing. The phase images show the morphology, and the total numbers of gravid parasites (white numbering) and of expected progenies (red numbering) from the current replication cycle have been labeled. The parasites with more than two daughter scaffolds are marked by yellow numbers. DNA (Hoechst)-staining images have been converted into gray scale for better visualization. The nuclei marked with green letters show symmetrical and the ones marked with blue letters show asymmetrical karyokinesis. (C, D) TgOTUD3A-KO parasites exhibiting aberrant endodyogeny in which the nuclear and budding cycles are uncoupled, resulting in completion of karyokinesis with the absence of cytokinesis (no daughter scaffold formation, yellow zero). In rare instances, single immature daughter scaffolds are observed where karyokinesis has been completed (yellow arrowhead). Scale bars = 10 µm. Download FIG S3, TIF file, 0.3 MB. Copyright © 2017 Dhara et al. 2017 Dhara et al. This content is distributed under the terms of the Creative Commons Attribution 4.0 International license.

In addition to instances where daughter scaffolds indicative of active cytokinesis were observed (Fig. 2B and C), the detection of karyokinesis (the presence of 2 [or more] discrete nuclei within a single mother parasite) with an absence of any visible daughter scaffolds (Fig. 2D) was indicative of an uncoupling of mitosis and cytokinesis that was typical of a schizogony-like replication strategy (5). Among these examples were instances where we appeared to have captured mitosis as it was occurring, seen as a dividing nucleus (Fig. 2D, DNA, green number 2). The unmarked nuclei in Fig. 2D appeared to be in parasites that were not cycling. Finally, the detection of a single immature daughter scaffold (Fig. S3D, TgIMC3, yellow arrowhead) in a mother parasite with completed mitosis within a vacuole where the other parasites had undergone or were in the process of mitosis indicated the potential for the aberrant initiation of cytokinesis at low frequency.

The deviations from normal endodyogeny were observed at higher frequencies in TgOTUD3A-KO parasites. We therefore plotted the distribution of parasite numbers within randomly acquired vacuoles counted on blinded samples. The results revealed the expected progression of growth for wild-type parasites (both RH and RH-TgOTUD3A-HA), with a distribution that follows the 2^*n*^ pattern and a small fraction of the vacuoles deviating from this progression (Fig. 2E). The identical analysis performed using TgOTUD3A-KO mutants revealed a significant increase (38%) in the proportion of non-2^*n*^ parasite-containing vacuoles (Fig. 2E). Notably, the proportions of vacuoles harboring 1 to 4 mutant parasites were not statistically different from the results for the wild type, but there was significant deviation at the 8-parasite stage (Fig. 2E). This suggested that the phenotype resulting in deviation from the 2^*n*^ growth progression manifested following the initial rounds of replication.

We also counted the numbers of daughters within gravid mother parasites by counting the numbers of daughter scaffolds (detected using TgIMC3 labeling) within them. Consistent with previously published data (39, 40) for actively growing wild-type parasites, 6.6% of mothers contained 3 daughter scaffolds (Fig. 2F). In contrast, the frequency of TgOTUD3A-KO mutant mother parasites with 3 daughters was 14.5% among gravid parasites (Fig. 2F). A similar proportional increase was observed for mothers bearing 4 daughter scaffolds (Fig. 2F), with a small number (1 out of 500 gravid parasites) containing 5 scaffolds (Fig. 2F). This suggested that the partial loss in the fidelity of the counting mechanism to ensure accurate endodyogeny (resulting in 2 progeny) was evident in the TgOTUD3A-KO organisms. Notably, this analysis did not include instances of the schizogony-like phenotype (Fig. 2D) or the presence of polyploid nuclei without daughter scaffolds (see discussion of electron microscopy [EM] results below), thereby underestimating the scope of deviations from endodyogeny within the TgOTUD3A-KO parasites. Notably, the phenotype of the independent clone 2C3 (data not shown) mirrored that of clone 2H1 detailed here.

In addition to examining the progression of mitosis and cytokinesis, we also examined the characteristics of the inheritance and formation of key organelles in mutant parasites. Multiple Golgi stacks were found in the mothers producing multiple daughters (Fig. S4), but in general, the numbers of apicoplasts and Golgi stacks matched the inferred numbers of daughter progeny based on the morphologies and intensities of the nuclei, suggesting faithful inheritance (Fig. S4 and S5). In addition, no defects were observed in the population with regard to the organization of the mitochondria, rhoptries, and micronemes (Fig. S6). These data suggested that the progeny resulting from the aberrant endodyogeny were normal and viable. This is consistent with the mutant out-competing the wild-type parasites in a head-to-head competition (Fig. 1F).

10.1128/mBio.01846-17.5 FIG S4  Organization of the Golgi bodies in mothers bearing >2 progeny. TgOTUD3A-KO parasites exhibiting multiple daughter scaffolds faithfully inherit Golgi bodies. Parasites were transiently transfected with a plasmid expressing the Golgi body structural protein GRASP55 fused to yellow fluorescent protein (YFP). (A) Transfected wild-type parasites show the normal Golgi stacks (YFP fluorescence) in parasites in G_1_ phase (single Golgi body). (B) Golgi bodies are duplicated and inherited early in cytokinesis. (C) TgOTUD3A-KO parasites that lack daughter scaffolds (TgIMC3) but have divided (or are in the process of dividing) their nuclei (DNA and DNA gray) exhibit aberrant proliferation of the Golgi bodies (GRASP55-YFP). Their nuclear morphology suggests that these might be on the path of producing multiple daughters. (D) TgOTUD3A-KO parasite containing 3 daughter scaffolds possesses an equal number of discrete Golgi stacks (GRASP55-YFP, yellow arrowhead). Notably, enlarged parasites (Phase) with likely abnormal polyploid nuclei (DNA and DNA gray) but no daughter scaffolds appear to have multiple or enlarged Golgi stacks as seen in panel C. TgOTUD3A-KO parasites with 2 daughter scaffolds were found to be similar to wild-type parasites (A and B). Scale bars = 10 µm. Download FIG S4, TIF file, 0.4 MB. Copyright © 2017 Dhara et al. 2017 Dhara et al. This content is distributed under the terms of the Creative Commons Attribution 4.0 International license.

10.1128/mBio.01846-17.6 FIG S5  Apicoplast levels in TgOTUD3A-KO mutants. TgOTUD3A-KO parasites faithfully inherit the apicoplast, consistent with their fitness advantage over wild-type parasites. Parasites were stained with an apicoplast marker antibody (TgAtrx1, green). (A) Wild-type parasites in both G_1_ and M/C phase (based on IMC3 [red] and DNA staining [gray scale]) exhibit normal localization, morphology, and inheritance of apicoplast. DNA staining (grayscale image) shows uniform nuclear division and morphology. (B) TgOTUD3A-KO parasite with multiple daughters shows equal numbers of apicoplasts, indicating that apicoplast division and inheritance is not significantly altered when multiple daughters are present. Scale bars = 10 µm. Download FIG S5, TIF file, 0.3 MB. Copyright © 2017 Dhara et al. 2017 Dhara et al. This content is distributed under the terms of the Creative Commons Attribution 4.0 International license.

10.1128/mBio.01846-17.7 FIG S6  Organization of micronemes, rhoptries, and mitochondria in TgOTUD3A-KO parasites. Immunofluorescence analysis of microneme (A), rhoptry (B), and mitochondrion (C) distributions in wild-type (WT) and TgOTUD3A-KO parasites reveals no gross aberrant phenotypes associated with the generation of these organelles. This suggests that the impact of TgOTUD3A-KO is primarily at the earlier stages of replication, affecting the number of progeny formed and not the maturation of progeny. Once committed to the initiation of cytokinesis, replication results in the proper inheritance or formation of these key organelles. In each instance, the specific organellar marker (microneme, TgMIC3; rhoptry, TgROP7; and mitochondrion, TgF1β) is labeled in green and the surface marker TgSAG1 in red. The parasite nucleus is labeled using Hoechst dye (DNA). Scale bars = 10 µm. Download FIG S6, TIF file, 0.6 MB. Copyright © 2017 Dhara et al. 2017 Dhara et al. This content is distributed under the terms of the Creative Commons Attribution 4.0 International license.

### Electron microscopy confirms the diverse patterns of replication in TgOTUD3A-KO parasites.

**FIG 3:**
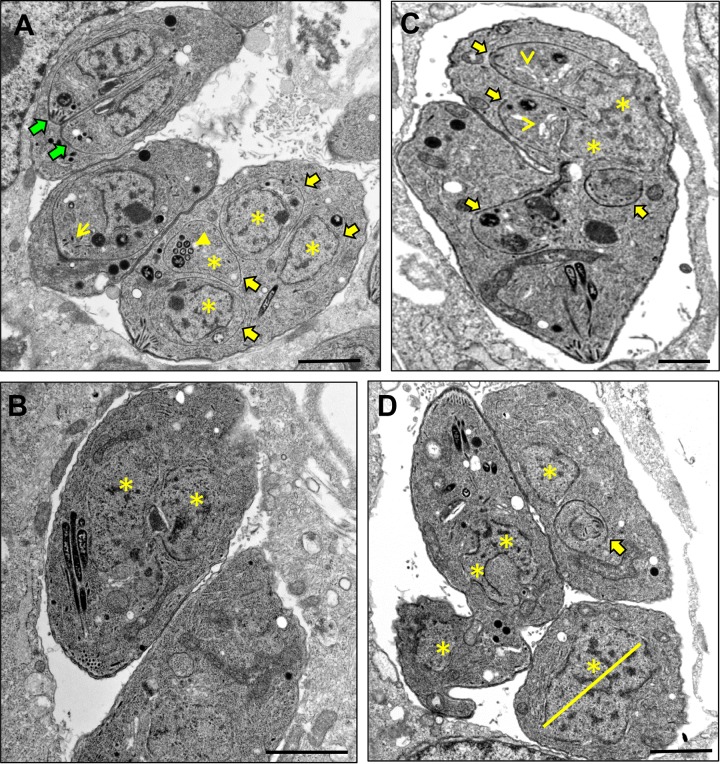
Replication patterns deviating from normal endodyogeny revealed by electron microscopy. Actively growing TgOTUD3A-KO parasites were prepared for electron microscopy and viewed with the aim of capturing evidence for deviations from normal endodyogeny. Normal endodyogeny, with 2 nearly mature daughter scaffolds within a TgOTUD3A-KO mother parasite, was detected (A; green block arrows) in the same vacuole shared with a mother having multiple daughter scaffolds (A, C; yellow block arrows) and multiple discrete nuclear profiles (B, C, D; yellow asterisks). A schizogony-like phenotype, the completion of mitosis without the initiation of cytokinesis (absence of daughter scaffolds), was also detected in a subset of mutant parasites (B; yellow asterisks). We see instances of what appears to be the reinitiation of a replicative cycle prior to the completion of a round of cytokinesis, presenting as 4 daughter scaffolds with 2 scaffolds in each arm of the aberrant parasite shown (C). Furthermore, the detection of large, likely polyploid nuclear masses without a daughter scaffold (D; yellow asterisk and line) is suggestive of an endopolygeny-like replication strategy. Parasites containing multiple daughter scaffolds and progressing through cytokinesis appear to possess normal micronemes (yellow-line arrow), rhoptries (solid yellow arrowhead), and Golgi bodies (open yellow arrowheads), consistent with the formation of these organelles observed by fluorescence microscopy (see Fig. S4 to S6 in the supplemental material). In all instances, the scale bars represent 1 µm.

In light of the significant deviations from conventional endodyogeny exhibited by the TgOTUD3A-KO, we sought to examine growing parasites by transmission electron microscopy (TEM). In addition to gravid mother parasites undergoing conventional endodyogeny, with 2 nuclei and 2 daughter scaffolds evident (Fig. 3A, green block arrows), mother parasites containing various numbers of daughter scaffolds were also observed in the same vacuole (Fig. 3A), consistent with an altered replication strategy that was found at higher frequencies in TgOTUD3A-KO parasites captured by immunofluorescence assay (IFA) (Fig. 2B to D). In the same vacuole, there was a parasite with a single scaffold visible in the section (an additional scaffold is likely in a distinct section) (Fig. 3A). The parasite shown in Fig. 3B represented a clear apparent example of the schizogony-like phenotype that was seen, as 2 distinct nuclei (Fig. 3B, yellow asterisks) were evident, indicative of completed karyokinesis with the absence of any cytokinesis (no daughter scaffolds). Although one cannot rule out the presence of scaffolds on a different EM section, it is unlikely due to the fact that we also captured this evidence by IFA (Fig. 2D). We further observed a parasite mass that appeared to contain 4 daughter scaffolds (Fig. 3C, yellow block arrows) and a dividing nucleus segregating between 2 progeny (Fig. 3C, yellow asterisks) in an overall configuration that appeared to indicate that 2 distinct rounds of mitosis might be occurring simultaneously or, alternatively, a new entry into cytokinesis was initiated without completion of the prior cycle. Finally, the vacuole shown in Fig. 3D was distinctive, as it contained a parasite containing a very large nucleus (Fig. 3D, yellow asterisk and line), much like an endopolygeny-like polyploid nucleus, as well as the parasite on the left, which exhibited a forked nucleus suggestive of ongoing karyokinesis that was curiously oriented away from the apical end, while its cytoplasm was connected to a fragment of another parasite.

Notably, examination at the ultrastructural level confirmed that the developing daughter parasites appeared to have morphologically normal micronemes (Fig. 3A, yellow-line arrow), rhoptries (Fig. 3A, closed yellow arrowhead), and Golgi bodies (Fig. 3C, open yellow arrowheads). In addition, mitochondrion and apicoplast profiles were also evident in parasites with aberrant endodyogeny that possessed morphological features of schizogony-like and endopolygeny-like replication architectures.

### The stoichiometry of the centrosome cores matches that of the daughter scaffolds.

**FIG 4:**
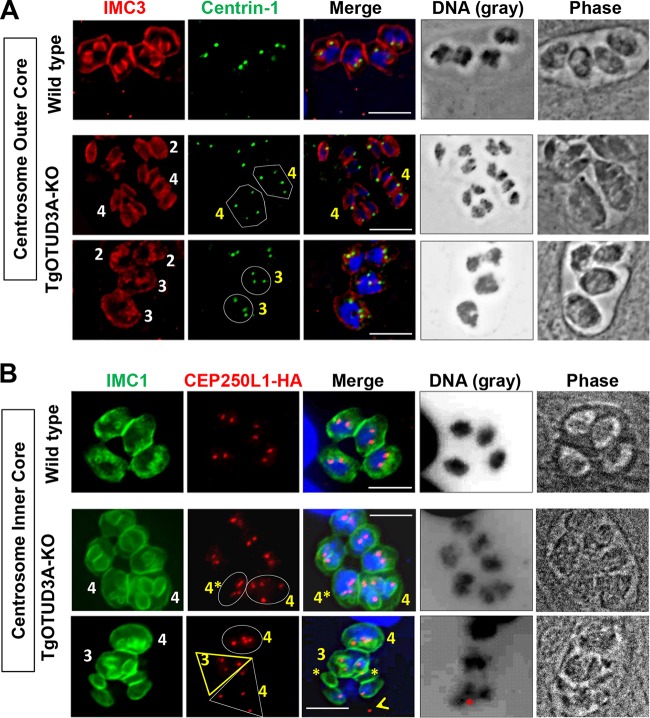
Centrosome numbers match the numbers of daughter scaffolds in gravid parasites. The bipartite *T. gondii* centrosome consists of an outer (Centrin-1 marker) and an inner (CEP250L1-HA marker) core. (A) (Top) Wild-type parasites, each with two daughter scaffolds (IMC3, red), possess duplicated centrosomes (Centrin-1, green) adjacent to the dividing nuclei. (Middle) In the gravid TgOTUD3A-KO parasites, mothers containing 2 daughter scaffolds have matched numbers of Centrin-1 spots (green, not marked). The gravid mothers containing 4 daughter scaffolds each (IMC3, red with white 4’s) are matched with regard to both Centrin-1 (green, marked with polygons) and discrete postmitotic nuclei (DNA and Merge). (Bottom) The parasites containing 2 daughter scaffolds (IMC3, red) each contain a single nucleus with 2 centrosomes (Centrin-1, green). Two larger mother parasites (Phase) with large polyploid nuclei (DNA) each contain 3 daughter scaffolds (IMC3, red with white 3’s), which are matched to 3 centrosomes (Centrin-1, green, circled). (B) (Top) In wild-type parasites containing 2 immature daughter scaffolds (IMC1, green), the numbers of centrosome inner cores (CEP250L1-HA, red) are matched and in close apposition to the intensely labeled undivided nuclei (DNA). (Middle) Mother TgOTUD3A-KO parasites containing the normal 2 daughter scaffolds (IMC1, green, not marked) are matched to the number of CEP20L1-HA (red)-labeled centrosome inner cores. Two additional parasites each contain four daughter scaffolds (IMC1, white 4’s) matched to 4 CEP240L1-HA (red) centrosome inner cores (circled). Each of these mothers possesses 2 equivalently labeled nuclei. (Bottom) Three TgOTUD3A-KO mother parasites containing 4 daughters (IMC1, green with white 4), 3 daughters (IMC1, green with white 3), and a rare failed replication event (IMC1, not numbered). Both the mothers containing 4 (circled) and 3 (marked with yellow triangle) daughters contain matched numbers of CEP250L1-HA-labeled centrosome inner cores. The nuclei in these parasites appear intensely labeled and polyploid (DNA and Merge). The apparently failed replication event contains 4 daughter scaffolds (IMC1) and 4 centrosome inner cores (CEP250L1-HA, red, marked with white triangle) but only 3 nuclei (DNA), one of which (Merge and DNA, red asterisk) is not associated with any daughter scaffold. In addition, two daughter scaffolds (IMC1 and Merge, yellow asterisk) lack a nucleus, while a free centrosome (Merge, open yellow arrowhead) appears to no longer be associated with either a nucleus or a scaffold. Scale bars = 10 µm.

As in higher eukaryotes, the centrosome in Apicomplexa is the main microtubule organizing center (MTOC) involved in coordinating mitosis and karyokinesis. A recent study (35) showed that the *Toxoplasma* centrosome has a unique bipartite structure (comprising outer and inner cores) that critically regulates the fidelity of the parasite cell cycle by regulating and integrating the dynamics of the nuclear and budding cycles. We therefore investigated whether the aberrant replication phenotypes seen in the TgOTUD3A-KO parasites were due to dysregulation of centrosome dynamics. Using an outer core protein (Centrin-1) and an inner core protein tagged with hemagglutinin (CEP250L1-HA) as markers, we examined the numbers and the distribution of centrosomes relative to the numbers of developing daughter scaffolds in gravid parasites, with a focus on mothers bearing more than two daughters (Fig. 4). The outer core is believed to associate with and regulate the spatial organization of the daughter scaffolds (35). As expected, for wild-type parasites undergoing mitosis (bilobed nuclear mass) with 2 immature daughter buds per mother, 2 Centrin-1 spots were associated with each nucleus/nuclear arm (Fig. 4A, wild type). The same was observed for the TgOTUD3A-KO parasites with 2 daughter scaffolds, whether the nuclei were divided (Fig. 4A, middle row, 2 scaffolds [IMC3] and 2 discrete nuclei) or undivided polypoid masses (Fig. 4A, bottom row, 2 scaffolds [IMC3] with 1 large nucleus each). In instances where more than 2 daughter scaffolds were detected in the mother, the number of Centrin-1 spots matched the number of scaffolds (Fig. 4A, TgOTUD3A-KO, Centrin-1, polygons and circles). Of note, the number of Centrin-1 spots within parasites containing very large nuclei but lacking any daughter scaffolds was found to be an indicator of the number of progeny that would be expected to develop.

The inner centrosome core is known to engage the intranuclear centrocone (which houses the mitotic spindle) (42, 43) to coordinate the outer core and ensure the fidelity of inheritance of the nuclear genome (35). Parasites in the later stages of S phase (inferred from the intensity of DNA staining) (Fig. 4B, Wild type, DNA) or initiating mitosis with the normal 2 immature daughter scaffolds possessed 2 CEP250L1-HA spots (inner core marker) associated with the nucleus (Fig. 4B, Wild type). This relationship was maintained for gravid TgOTUD3A-KO parasites bearing 2 daughter scaffolds both with an undivided nucleus and with already-separated nuclei following karyokinesis (Fig. 4B, TgOTUD3A-KO). As was observed with the outer core marker Centrin-1, the number of CEP250L1-HA-positive spots matched the number of daughter scaffolds whether the mother bore 3 daughter scaffolds (Fig. 4B, CEP250L1-HA, bottom row, bold yellow triangle) or 4 daughter scaffolds (Fig. 4B, CEP250L1-HA, middle and bottom rows, ovals). These data indicated that at the time cytokinesis was initiated (appearance of daughter buds), the numbers of scaffolds, outer cores, and inner cores were in a 1:1:1 ratio. In rare cases, we observed that the progeny of multiple-daughter parasites were present as scaffolds lacking nuclei (Fig. 4B, Merge, bottom row, yellow asterisks). In this instance, a nucleus (Fig. 4B, DNA, bottom row, red asterisk) appeared outside its parent parasite with a rogue centrosome that was no longer associated with it (Fig. 4B, bottom row, white triangle and open yellow arrowhead). These data clearly show that parasites gravid with multiple daughters, captured early in the budding cycle (based on the small immature daughter buds) at the onset of cytokinesis, are competent to produce viable progeny.

### Transient dysregulation in the stoichiometry of the inner and outer cores of the centrosome drives deviation from endodyogeny.

Ploidy within apicomplexan parasites undergoing different modes of replication is controlled at the level of the centrosome (35). Deviations from a ploidy of 2 (endodyogeny) in TgOTUD3A-KO parasites are noted by the centrosome number matching that of the daughter scaffolds as gravid parasites enter cytokinesis (Fig. 4). This suggests that any aberration during centrosome duplication (as would be required for the generation of more than 2 progeny) would have to occur by an aberrant reduplication of one (to result in 3 progeny) or both (resulting in 4 progeny) centrosomes. These changes would likely be reflected as transient imbalances in centrosome dynamics during the phase of DNA synthesis, serving to alter S-phase progression while ensuring the completion of the required ploidy to match the eventual number of daughters. A failure to match the ploidy to the number of daughters would result in catastrophic defects that would be inconsistent with the enhanced fitness of TgOTUD3A-KO mutants (Fig. 1) (38).

**FIG 5:**
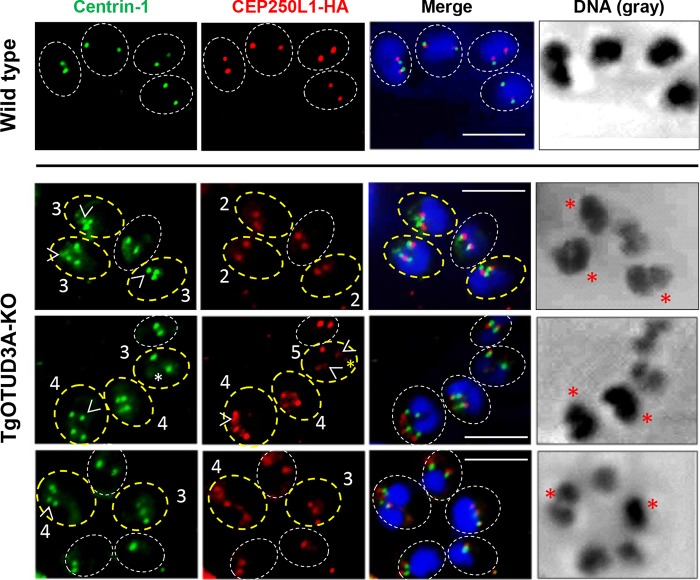
Reduplication and transient dysregulation in the stoichiometry of the inner and outer cores drive the plasticity of the replication mode. The 1st row displays wild-type parasites both primed for mitosis (based on nuclear size and intensity) and undergoing mitosis, possessing matched outer (Centrin-1, green) and inner (CEP250L1-HA, red) cores in close apposition to each other and the nucleus (Merge). The 2nd and 3rd rows show the diverse phenotypes associated with the TgOTUD3A-KO mutant. These include instances of two matched inner and outer centrosome cores (circled in white dashed lines), similar to the phenotype of wild-type parasites. TgOTUD3A-KO parasites with more than 2 inner or outer cores are circled in yellow dashed lines. These include parasites within which the number of outer cores (Centrin-1) exceeds the number of inner cores (CEP250L1-HA); the extra cores are marked with open white arrowheads. The 1st row for the TgOTUD3A-KO line displays three parasites containing mismatched outer and inner cores. These parasites display aberrant nuclear morphologies (red asterisks), with two nuclei presenting as larger masses (9 o’clock and 11 o’clock) relative to the sizes of the mitotically resolved nuclei within the normal parasite (circled in white dashed lines). The third parasite (5 o’clock) appears to have undergone one round of mitosis but displays unevenly sized nuclei. The 2nd row for the TgOTUD3A-KO line displays a parasite with both higher numbers and mismatch (in terms of their relative positioning) of both outer and inner cores (Centrin-1, 7 o’clock) that possesses 2 intensely labeled nuclei of equivalent size. In addition, this vacuole contains a parasite with additional inner cores (CEP250L1-HA, 2 o’clock). In this case, the weak signal of an outer core (Centrin-1, white asterisk) and some inner cores (CEP250L1-HA, yellow asterisk) may suggest an immature centrosome or breakdown products of a centrosome. Of note, the parasite in the center (5 o'clock) possesses 4 matched Centrin-1- and CEP250L1-HA-labeled centrosomes and appears, based on the intensity of the DNA synthesis, closer to the end of replication and poised for the second round of symmetrical mitosis and cytokinesis. Of the two TgOTUD3A-KO parasites displayed in the 3rd row with more than 2 centrosome cores, one (10 o’clock) possesses matched though spatially disorganized centrosomes together with 2 largely matched nuclei. The second aberrant parasite (3 o’clock) exhibits an intensely labeled apparently polyploid nucleus within which the stoichiometry between the inner and outer cores appears to have been resolved. The resolution of transient imbalances in favor of either the inner or outer core appears to be linked to an aberrant S phase, promoting either a schizogony-like or endopolygeny-like nuclear cycle. In all instances, the scale bars represent 10 μm.

Duplication of the bipartite centrosome during endodyogeny followed a prescribed pattern that was initiated with the duplication of the outer (Centrin-1) core prior to that of the inner (CEP250L1-HA) (35) core. The completion of normal centrosome core duplication during endodyogeny resulted in a 1:1 stoichiometry with the cores in close apposition to each other. The association of the inner core with the centrocone (spindle) coupled with the attachment of the DNA constituted the priming for mitosis. Toward establishing whether there were transient defects in the stoichiometry of the centromere cores that were corrected prior to entry into cytokinesis, we examined the stoichiometry of the centrosome cores using the Centrin-1 and CEP250L1-HA signals. These patterns were correlated to the size, shape, and intensity (a measure of total DNA content) of the relevant nuclei (Fig. 5). Accordingly, the wild-type parasites (Fig. 5, Wild type) appeared to be in the midst of mitosis, typified by a larger nucleus, likely at the end of S phase based on the high concentrations of DNA and the distribution of inner and outer cores (Fig. 5). For these 4 wild-type parasites, there was a 1:1 stoichiometry between the outer core (Centrin-1) and inner core (CEP250L1-HA), with the 2 signals found to be associated with the nucleus and in close apposition to each other (Fig. 5, Wild type, Merge). When examining the TgOTUD3A-KO mutant parasites, we often detected 3 or more centrosomal cores (outer and/or inner) in a subset of parasites (Fig. 5, TgOTUD3A-KO parasites circled with bold yellow dotted lines) within a vacuole, indicating a deviation from normal endodyogeny. Those circled with white dotted lines in Fig. 5 appeared to be executing conventional endodyogeny. Notably, as seen with the daughter scaffolds (Fig. 2), parasites within the same vacuole (phase image, not shown) exhibit features of classical endodyogeny and significant variation from this replication strategy. More often we saw instances where the outer core number exceeded the inner core number (Fig. 5, TgOTUD3A-KO, top row, 4 o’clock, 9 o’clock, and 11 o’clock positions, white open arrowheads). A lower frequency was observed for instances where there were more inner cores than outer cores. An example of this phenotype was evident within a vacuole where one parasite possessed 3 outer cores and 5 inner cores (Fig. 5, TgOTUD3A-KO, middle row, 2 o’clock position). Besides observing the imbalance, we also captured the instances where 3 or more outer or inner cores were in perfect 1:1 stoichiometry and sitting in close appositions (Fig. 5, TgOTUD3A-KO, middle row, 7 o’clock and 5 o’clock positions, and bottom row, 10 o’clock and 3 o’clock positions). This suggested that 1:1 stoichiometry could be or is achieved in these TgOTUD3A-KO parasites even though there is a transient loss of core stoichiometry. Upon the restoration of inner and outer core stoichiometry, we expected that entry into the budding cycle (cytokinesis) was now permissible.

Next, we investigated whether this transient asymmetry in the centrosome cores drives aberrant DNA replication (more than 2N ploidy), thereby altering the nuclear cycle in the TgOTUD3A-KO parasites. The grayscale images of nuclei in the TgOTUD3A-KO parasites containing multiple inner or outer core spots revealed that a prominent imbalance in the stoichiometry or the presence of more than 2 centrosome cores was often associated with abnormal nuclear morphologies, seen as significantly larger nuclei with more than 2N nuclear mass (Fig. 5, TgOTUD3A-KO, DNA, red asterisks), suggesting that there was failure to terminate S phase at 2N ploidy and the excess centrosome drove the DNA replication to continue until the correct ploidy was achieved for successful entry into the budding cycle. These findings provide a mechanistic basis underlying the multiple-daughter phenotype and the potential for schizogony-like and endopolygeny-like nuclear cycles evidenced by IFA (Fig. 2 and 4) and electron microscopy (Fig. 3).

### Incomplete centrosome engagement with a partner centrocone (spindle) promotes an altered progression of the nuclear cycle.

**FIG 6:**
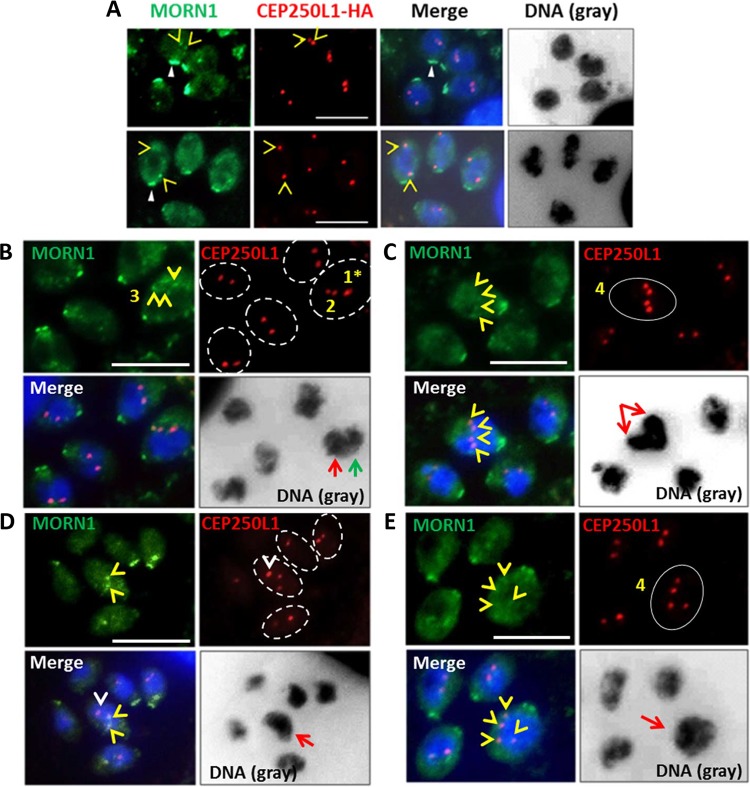
Centrocone (MORN1) and centrosome dynamics define the mechanistic basis for the production of multiple progeny. MORN1 is a marker of the nuclear centrocone (open yellow arrowheads), as well as the basal complex (closed white arrowheads). (A) In the wild-type parasites, the centrocone is in close apposition to the centrosome inner core (CEP250L1-HA). The images in the top and bottom rows exhibit the association of the centrocone with the centrosome in early and late S phase, respectively, which facilitates engagement of the genome with the centrosome. A balanced stoichiometry between the centrocones and centrosomes facilitates mitosis and karyokinesis. (B) While 4 of the parasites in the vacuole possess correctly organized centrosome-centrocone pairs, the fifth organism appears to have reduplicated one centrosome (CEP250L1-HA, DNA, red-line arrow) and the other remains poised for division. (A and B) In both panels, second reduplications of one or both centrocone-centrosome pairs appear to be happening in the nuclei that have undergone first-round karyokinesis, similar to a schizogony-like replication strategy. (C) Two perpendicularly arranged nuclei exhibiting the reduplication of the centrosome inner core (CEP250L1-HA) appear matched with the centrocone marker MORN1 (open yellow arrowheads), suggesting that they are capable of mitosis. Note the intensity of the nuclear signal, suggestive of more DNA content than in 1N ploidy. (D) The parasite in the center of the field exhibits two nuclear MORN1-labeled centrocones (open yellow arrowheads) but 3 distinct centrosome inner cores (CEDP250L1-HA). Notably, the orphan centrosome (open white arrowhead) renders it incompetent for the engagement of the spindle checkpoint. The nucleus in the parasite (red-line arrow) appears to be a large and polyploid nuclear mass. (E) Experimental evidence also demonstrates that one gravid parasite that has four inner core spots (CEP250L1-HA, red, circled in white) and four matching centrocone spots (MORN1, green) contains a very large polypoid nucleus (DNA, red arrow), suggesting that they are primed for making 4 progenies. (D and E) Both patterns of labeling are suggestive of an endopolygeny-like replication event in process, as in both cases, mitosis may have been completed even though karyokinesis is yet to happen. Scale bars = 10 µm.

**FIG 7:**
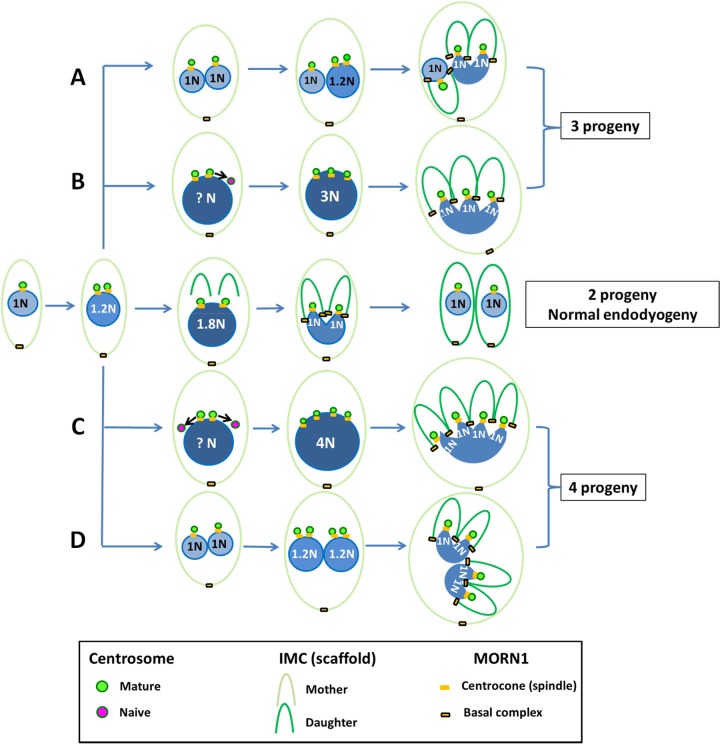
Schematic models for the generation of multiple progeny in the TgOTUD3A-KO line. (Middle) Schematic displays the process of normal endodyogeny, highlighting key milestones with regard to both the nuclear and budding cycles. With conventional endodyogeny, an integrated nuclear (DNA synthesis and mitosis, karyokinesis) and budding (formation of daughter scaffold) cycle progresses to generate 2 daughters. (A, B) Deviations from endodyogeny in TgOTUD3A-KO parasites resulting in the formation of three daughter progeny by both an endopolygeny-like and a schizogony-like mechanism due to an asymmetrical division of the genome(s) during the nuclear cycle. (C, D) Generation of four daughter parasites using both a schizogony-like and an endopolygeny-like mechanism, driven by the symmetrical reduplication of the genome. These deviations from conventional endodyogeny are driven by aberrant reduplication of the centrosome and the capacity of the new centrosomes to engage partner centrocones. Incomplete centrosome-centrocone pairing prevents the arrest of DNA synthesis, as the spindle checkpoint cannot be engaged (see text for more details).

The most critical function of the centrosome is to anchor the mitotic spindle at the kinetochore to drive mitosis and karyokinesis. In light of mitosis in *Toxoplasma* being closed (the nuclear envelope does not disintegrate as the chromosomes separate), a specialized structure termed the centrocone (42–44) is required to establish access for the cytoplasmically located centrosomes to the chromosomes in the nucleus. The presence, therefore, of multiple centrosomes in multiply gravid TgOTUD3A-KO parasites would necessitate the formation of matched centrocones to ensure the correct engagement of the respective genome equivalents to the centrosomes, ensuring the fidelity of inheritance. This issue is particularly relevant in instances where a single polyploid (>2N ploidy) nuclear mass is present. To test this, we used an antibody against membrane occupation and recognition nexus protein 1 (MORN1), a protein marker of the centrocone (Fig. 6, yellow open arrowheads and closed white arrowheads) (42, 43), in the CEP250L1-HA-tagged TgOTUD3A-KO line to establish the relationship between the centrosome and centrocone. Importantly, the MORN1 protein is found not only in the centrocone (Fig. 6, yellow arrowhead, and Fig. 7) but also at the apical (not shown) and basal ends of the parasite (Fig. 6A, white closed arrowhead, and Fig. 7, Normal endodyogeny) (42, 43). As the parasite completes G_1_ and enters S phase, DNA synthesis proceeds along with the duplication of the centrosomal cores and the centrocone also duplicates, establishing its association with the centrosome inner core (Fig. 6A and 7, Normal endodyogeny). When the ploidy reaches 1.8N, the newly duplicated chromosomes associate with the centrosome through the centrocone, which functions as the conduit (Fig. 7, Normal endodyogeny). This effectively engages the spindle checkpoint to terminate DNA synthesis (S phase), initiating mitosis and karyokinesis. This proceeds with the increase in the distance between centrosome pairs as the nucleus divides (Fig. 6A, bottom row, open yellow arrowheads, and Fig. 7, Normal endodyogeny) (3). Given the overlapping organization of the cell cycle, the process of formation of daughter buds is initiated before mitosis is complete (Fig. 7) (5, 10). As mitosis proceeds, the daughter scaffolds (IMC signal) extend (Fig. 7, Normal endodyogeny) and connect at their leading edges to MORN1, which will form the eventual basal complex in the daughter parasites (Fig. 6A, closed white arrowheads, and Fig. 7, Normal endodyogeny) (5, 10, 42).

The altered progression of endodyogeny resulting in the generation of 3 or 4 (or more) daughter parasites appeared to be associated with two different mechanisms revealed by the TgOTUD3A-KO mutant parasites. The first is uncoupling of the nuclear and budding cycles due to reduplication of one or both of the centrosomes associated with the nuclei that have completed the first nuclear cycle (schizogony-like) (Fig. 6B and C and 7A and D). Instead of moving into the budding cycle, one or both individual nuclei (Fig. 6B and C, red-line arrows) undergo further duplication to match the centrosome number, resulting in 3 and 4 progeny, respectively. In each of these instances, the nuclei that are undergoing a second round of division are either larger or denser than the 1N nucleus (Fig. 6B and C, red-line arrows, and Fig. 7A and D).

The second mechanism is attributable to the failure of S phase to terminate upon reaching 2N ploidy due to the reduplication of one or both centrosomes, resulting in a 3N or 4N polypoid nucleus (endopolygeny-like) that finally resolves into 3 or 4 progeny (Fig. 6D and E and 7B and C).

We were able to capture the orphan centrosome (Fig. 6D, white arrowhead, and Fig. 7B and C, purple) waiting to receive its partner centrocone. This centrosome is expected to receive its partner centrocone (MORN1) upon completion of 3N ploidy. The absence of a MORN1 signal suggests that the spindle checkpoint cannot be engaged at this orphan centrosome. This absence of MORN1 attachment and the larger nuclear morphology and higher nuclear intensity (compared to that of adjacent parasite with 2 centrosomes) of these nuclei (Fig. 6D and E, red-line arrows, and Fig. 7B and C) suggest that these nuclei are still undergoing S phase. Together, these data support the idea that the centrosome dynamics and its association with spindle checkpoints drive the generation of multiple-daughter phenotypes in TgOTUD3A-KO parasites.

### Failure of complementation upon restoration of TgOTUD3A to the KO mutant.

**FIG 8:**
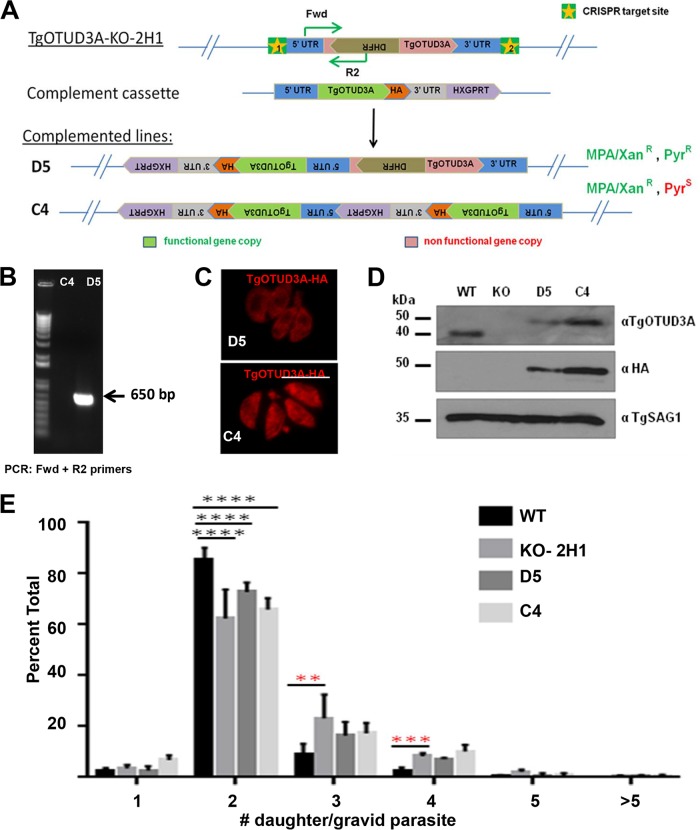
Complementation of TgOTUD3A-KO parasites does not appear to restore wild-type phenotypes. (A) Schematic shows the complementation strategy using the CRISPR-Cas9 gene-editing approach. Two CRISPR sites were designed to flank the 5′ and 3′ UTRs of the TgOTUD3A locus in the KO-2H1 strain, which has a knock-in mutated DHFR cassette. The complementing gene copy has its own endogenous promoter and C-terminal 3×HA tag and a functional HXGPRT cassette to provide mycophenolic acid/xanthine resistance (MPA/Xan^r^). Two complemented clones, which were selected based on resistance to MPA/Xan (clone D5 retains pyrimethamine resistance [Pyr^r^], and clone C4 is sensitive to pyrimethamine [Pyr^s^]), were used for further experiments. (B) The presence or absence of the DHFR cassette in those complemented lines was further confirmed by PCR (primer location is indicated in the schematic in panel A). (C) Immunofluorescence analysis with anti-HA antibody exhibits a higher level of TgOTUD3A expression in C4. Scale bars = 10 µm. (D) Immunoblotting with anti-TgOTUD3A and anti-HA antibodies confirms TgOTUD3A expression in the complemented lines and also at higher levels in C4 than in D5. (E) Comparison of multiple-daughter phenotypes between the WT, TgOTUD3A-KO, and 2 complemented lines (D5 and C4). Parasites were stained with anti-TgIMC3 antibody (to visualize daughter scaffolds) in gravid parasites (SAG1 labels individual parasites) and were blindly counted by two individuals (A.D. and A.P.S.). One-way ANOVA was done (F_22,63_ = 3.874, *P* < 0.0001), and the number of daughters per gravid parasite compared between any two groups using Tukey’s pairwise multiple-comparison test (α = 0.05). Significant differences (*P* ≤ 0.05) between groups are marked by asterisks as follows: **, *P* ≤ 0.01; ***, *P* ≤ 0.001; ****, *P* ≤ 0.0001. The data analyzed were from 4 biological replicate experiments. In total, 3,819 wild-type, 2,319 KO, 1,841 D5, and 1,664 C4 parasites were counted. Error bars represent standard deviations.

In order to confirm that the disruption of TgOTUD3A was responsible for the increased frequency of the multiple-daughter phenotype, we sought to restore the wild-type gene under the control of its native promoter at its original chromosomal location. The approach relied on the use of 2 CRISPR sites to drop out the mutagenizing (DHFR-containing) cassette and replace it with a C-terminally HA-tagged complementing cassette that included a hypoxanthine-guanine phosphoribosyltransferase (HXGPRT) gene to afford mycophenolic acid/xanthine (MPA/Xan) resistance in the ΔHXGPRT background of the strain (Fig. 8A). The resulting complemented clones were selected for MPA/Xan resistance and further screened for resistance or sensitivity for pyrimethamine to establish the retention or replacement of the original DHFR/thymidylate synthase (TS) resistance cassette. Two complemented clones of the 2H1 line, D5 (retaining both the mutagenized gene and a single copy of the complementing cassette encoding MPA/Xan^r^ and Pyr^r^; also designated Compl-2H1 below) and C4 (containing a tandem insertion of the complementing cassette and eliminating the mutagenized gene encoding MPA/Xan^r^ and Pyr^s^) were chosen for further analysis (Fig. 8A). The retention of the DHFR/TS cassette in the D5 clone was confirmed by PCR (Fig. 8B), and the restoration of the functional TgOTUD3A gene in both D5 and C4 was validated using immunoblotting and IFA in addition (Fig. 8C and D). Consistent with the presence of an additional copy of TgOTUD3A, complemented line C4 expressed higher levels of the protein than D5, as captured by both immunofluorescence (Fig. 8C) and immunoblot (Fig. 8D) analysis. The increase in the size of the complementing gene was due to the presence of the 3×HA epitope tag (Fig. 8D).

The ability to restore TgOTUD3A to the KO mutant was assessed using the multiple-daughter progeny phenotype counted on double-blinded samples. Remarkably, despite the expression of nearly wild-type levels (clone D5) or overexpression (clone C4) of TgOTUD3A, restoration of the wild-type phenotype was not achieved (Fig. 8E). Rather, the frequencies of multiply gravid mother parasites within the complemented lines were at the levels seen in the TgOTUD3A-KO parasites. In addition, the complemented line behaved exactly like the TgOTUD3A-KO line in the plaque assay (data not shown). At face value, this suggested that the multiple-daughter phenotype was due to an off-target mutation of a gene(s) other than TgOTUD3A. Alternatively, the loss of TgOTUD3A established an irreversible change as a compensatory mechanism, whereby the restoration of the gene had no effect on what was now the “new normal.”

### Whole-genome sequencing fails to identify a credible second site mutation responsible for the phenotype.

The inability to complement the defect in the TgOTUD3A-KO line (clone 2H1) suggested that a second site mutation potentially caused by the mistargeting of CRISPR-Cas9 or other means was responsible for the observed multiple-daughter phenotype. We reasoned that if this were the case, the potential off-target mutation would also be found in the independently isolated clone 2C3 and would be shared with the complemented 2H1 line (Compl-2H1 [clone D5]). In order to directly assess this, we performed whole-genome Illumina sequencing on a parental line (WT, RHΔHXGPRT), KO clones 2C3 and 2H1, and the Compl-2H1 line (D5). Details regarding the assembly and analysis of the NGS data are described in Text S1A in the supplemental material.

10.1128/mBio.01846-17.1 TEXT S1  (A) Detailed description of whole-genome-sequencing workflow and analysis. (B) One-way ANOVA of variants from whole-genome-sequencing data to establish the probability of identifying random mutations across the four sequenced lines. (C) List of identified SNPs across all four sequenced parasite lines on a per-scaffold basis. (D) Detailed analysis of the changes to amino acid/protein sequences due to the mutations within predicted open reading frames. Download TEXT S1, PDF file, 0.2 MB. Copyright © 2017 Dhara et al. 2017 Dhara et al. This content is distributed under the terms of the Creative Commons Attribution 4.0 International license.

**FIG 9:**
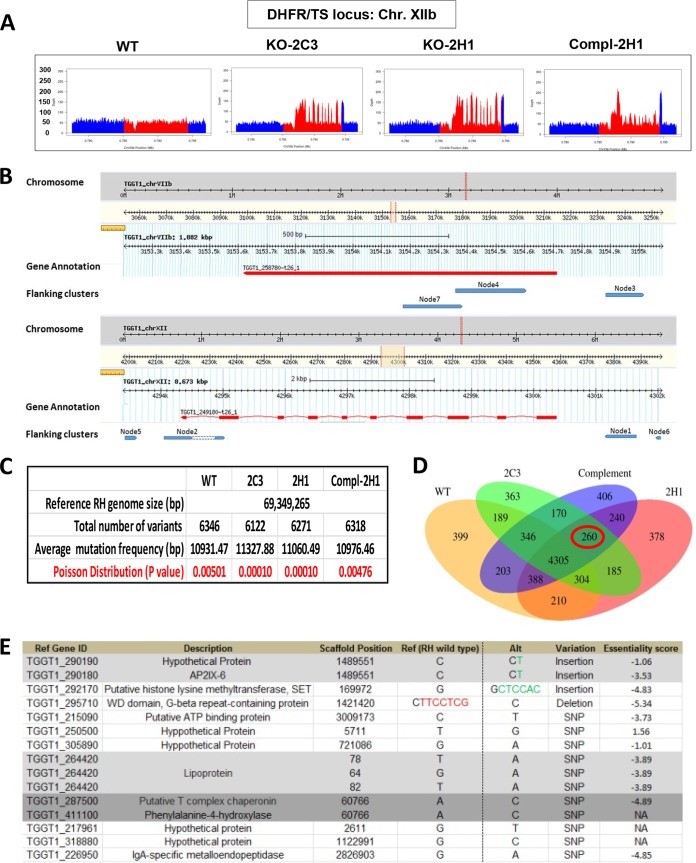
Whole-genome next-generation sequencing of 4 parasite lines (WT, 2 KOs, and a complemented line). (A) Each chart shows the read depth coverage of a portion of the *T. gondii* chromosome XIIb, where the red region represents the DHFR/TS locus, showing 3 copies of DHFR in the TgOTUD3A-KO samples (clones 2C3 and 2H1) and 2 copies of DHFR in the complemented 2H1 (Compl-2H1 [clone D5]) samples, compared to 1 in the wild type. (B) Analysis of the locations of the extra DHFR copies in the TgOTUD3A-KO and complemented lines. Flanking sequences of the drug cassettes in the top panel of the genome browser (derived from ToxoDB) output represent the TgOTUD3A locus, where 3 of the 7 hits match, and the bottom panel shows the DHFR locus, where the remaining 4 of the 7 hits aligned, indicating that the drug cassettes integrated in tandem in the TgOTUD3A locus without random insertion in the genome. (C) Statistical analysis shows that the average frequencies of mutation (based on the total number of variations and RH genome size) found in each sample and between samples are random and not caused by CRISPR editing. (D) Venn diagram comparing all variants called by our analysis, showing that 260 of them (circled in red) were shared between the mutants and not with the wild type. (E) Further analysis determined that out of 260 variants, only 15 were found inside the coding regions; these are listed in the table. Since there is no RH annotation available in the database, we used the GT1 gene identification number (ID) as the reference gene. Position, type of mutation, and essentiality score based on a recent genome-wide CRISPR study (38) are provided.

Genomic DNA libraries from 4 clonal lines were made using the TruSeq DNA PCR-free method and sequenced by Illumina HiSeq 2500 rapid-run sequencing, whereby 100- to 125-nucleotide (nt)-long fragments were sequenced. We obtained excellent average coverage of the genomes (WT, 53×; 2H1, 43×; 2C3, 40×; and Compl-2H1 [D5], 46×). This depth of coverage was evident for the number of reads at the DHFR/TS locus, which was doubled for the exons (as the mutagenizing cassette encodes the cDNA and not the genomic copy of the gene) of DHFR/TS in the 2 KO clones, resulting in a series of peaks for those strains (Fig. 9A, red peaks). The peak height for the DHFR/TS exons in the complemented line was double that of the wild type but two-thirds of that for the 2 KO lines, indicating that one of the 2 DHFR/TS cassettes was removed during complementation. In the Compl-2H1 (D5) strain, one of the two DHFR/TS cassettes was replaced with the epitope-tagged TgOTUD3A-HA and the HXGPRT selection marker. The higher red and blue peaks at the 5′ and 3′ ends for the complemented line, at levels similar to those for the KO lines, reflected that the same 5′ and 3′ untranslated regions (UTRs) of DHFR were used in the HXGPRT gene cassette as in the flanking sequences (Fig. 9A).

Because more than one DHFR/TS cassette got inserted into 2 independent KO lines, 2C3 and H1, we investigated whether those extra copies of the cassette were inserted in tandem at the TgOTUD3A locus or in a different locus other than the CRISPR-Cas9 target site (in this case TgOTUD3A), thereby resulting in an off-target insertional mutation. The recovery of DNA sequences flanking the mutagenesis cassette flanking region revealed that all 7 nodes captured by homology to the flanking 50 nt on both ends of the DHFR/TS cassette matched either the TgOTUD3A gene or the DHFR cassette itself, consistent with the tandem insertion of the DHFR cassettes at the TgOTUD3A locus (Fig. 9B).

We next sought to establish all the differences identified on our sequenced clones relative to the recently published *Toxoplasma gondii* RH reference genome (45). The RH genome showed excellent concordance (98% identical) with the reference type I GT1 sequence (http://www.toxodb.org) (Fig. S7). Using the published *Toxoplasma* RH genome as the reference, we identified between 6,122 and 6,346 variations in the wild-type (WT), mutant (2C3 and 2H1), and complemented (Compl-2H1) lines (Fig. 9C). Given the size of the published genome, this corresponded to an averaged frequency of a mutation from the reference genome roughly every 11,000 bases. The Poisson distribution *P* value for each of these samples, in the range of 0.00476 to 0.00010, served as a measure of a random occurrence not driven by a specific factor(s) (Fig. 9C). We also tested the significance of the observed variance between strains by single-factor analysis of variance (ANOVA) (Text S1B). Neither statistical analysis identified these variations as any different from random variations. Not surprisingly, the vast majority of these deviations from the reference genome (GT1; 4,305 differences) were shared among all the sequenced strains (Fig. 9D).

10.1128/mBio.01846-17.8 FIG S7  Alignment of published sequences for type I RH versus the reference type I GT1 genome. (A) RH and GT1 genome assemblies were aligned using the MuMmer aligner tool with a threshold setting of 70% identity. Each filled squared region represents the portion of the assembly that is equivalent to a scaffold in each genome assembly. The red colored lines denote same-strand alignment, while blue lines represent opposite-strand alignments between two genomes. This analysis also shows that there may be a few structural variations between these genomes, possibly caused by some gene rearrangements, but the genomic content seems to be complete, without any large deleted regions. (B) Whole-genome identity analysis reveals that the RH genome and the GT1 reference genome used in this study are 98.25% identical on average, with no low-identity region observed (minimum of >75%). Download FIG S7, TIF file, 13 MB. Copyright © 2017 Dhara et al. 2017 Dhara et al. This content is distributed under the terms of the Creative Commons Attribution 4.0 International license.

We next established the frequency of shared deviations from the reference genome in the 2 mutants and the complemented line (2C3, 2H1, and Compl-2H1 [D5]) that were not present in the parental parasites (WT). This population had 260 deviations from the reference genome (Fig. 9D, red circle), which were manually curated to eliminate deviations that were present in intragenic regions and within predicted introns. The survivors of this analysis comprised shared deviations within predicted open reading frames and were found to comprise insertions, deletions, and single-nucleotide polymorphisms (SNPs) (Fig. 7E; Text S1C). We sought therefore to assess the impact of loss-of-function mutations of these genes by exploiting the results from a recent whole-genome CRISPR knockout analysis (38). In this competition-based study, a score of 0 indicates no impact on growth, a negative score indicates a fitness defect, and a positive score is indicative of a growth advantage as a result of a loss of the targeted activity. Notably, these essentiality scores were based on the relative depletion or enrichment of CRISPR-disrupted genes over 3 lytic cycles, representing a relatively short time frame. As these TgOTUD3A-KO or complemented lines have been passaged for multiple lytic cycles and did not show any gross detrimental growth abnormality, these loci can be excluded as being the basis of the phenotype. Thus, any fitness defect or advantage would be expected to be exaggerated further with increasing lytic/passage cycles if these phenotypes were inherited. Each integer (positive or negative) reflects a 2-fold difference (log_2_) in growth. Of the genes for which essentiality scores were available, 8 resulted in significant growth defects (essentiality scores of −3.53 to −4.89) and 2 had a roughly 2-fold effect on growth (essentiality scores of 1.01 and 1.06) (Fig. 9E). This predicted negative impact on growth would exclude these genes as being responsible for the defect attributed to the TgOTUD3A-KO line, where the mutant parasites have a growth advantage (Fig. 1E). A single predicted gene presented a growth advantage (essentiality score of 1.56), indicating a roughly 3-fold increase in representation within the population over the 7-day-infection protocol used in the genome-wide KO study. We could safely eliminate this hypothetical gene from consideration, as extrapolation of the 2-fold difference in head-to-head competition between the wild-type and the TgOTUD3A-KO mutant at 48 h (Fig. 1E) would outstrip a 3-fold increase if extended to 7 days.

Finally, the 3 targets that were not included in the whole-genome CRISPR screen all had SNPs predicted to result in amino acid substitutions (Fig. S6D). The predicted phenylalanine-4-hydroxylase (TGGT1_411100), involved in the production of tyrosine from phenylalanine, presented with a substitution of R for L at position 772 (L772R). A recent study showed that ME49 tachyzoites with this gene homolog knocked out had no significant growth defect (46). And last, the substitutions in 2 hypothetical open reading frames (ORFs) TGGT1_217961 (L110I) and TGGT1_318880 (E668Q) were more conservative and unlikely to result in a loss of function. In addition, the nature of the phenotype was stochastic and it only appeared in some parasites within a given a vacuole, which indicated that a single amino acid substitution was not responsible. Taken together, we were unable to identify any credible shared mutation that could provide the basis of an off-target effect at the level of the genome that accounted for the phenotype exposed by the loss of TgOTUD3A. We therefore sought to establish whether the loss of TgOTUD3A triggered a compensatory change that irreversibly altered the expression landscape, thereby precluding the complementation of the lesion.

### Selective overexpression of closely related TgOTU family members in the TgOTUD3A-KO line.

**FIG 10:**
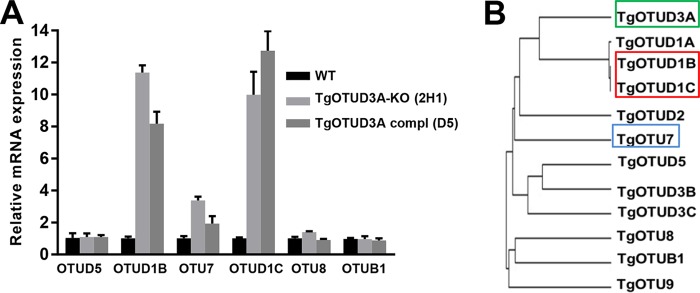
Members of the TgOTU family are selectively upregulated in TgOTUD3A-KO parasites, and complementation did not restore wild-type expression. (A) Knockout of TgOTUD3A results in the selective upregulation of specific TgOTUs. Steady-state mRNA expression of unsynchronized wild-type and TgOTUD3A-KO parasites infected for 24 h was quantified using real-time quantitative PCR. Data from quadruplicate samples from 3 independent experiments were analyzed by the ΔΔ*C*_*T*_ method after being normalized against TgSAG1 expression (template control). The mRNA expression of TgOTU7, one of the other 2 cell cycle-associated TgOTUs (TgOTU5 and TgOTU7), was increased by more than 3-fold. Significantly, the expression of two members of clade D1 TgOTUs (TgOTUD1B and TgOTUD1C) was upregulated 10- to 12-fold. Complemented line D5 (which has a single functional copy of the TgOTUD3A gene) showed an expression profile similar to that of the KO parasites, suggesting a failure of functional complementation even though the complemented gene copy was expressed in a dynamic pattern similar to the expression in the wild type. (B) Phylogenetic analysis revealed that the two significantly upregulated D1 TgOTUs (red box) are phylogenetically among the closest homologs of TgOTUD3A (green box). The position of TgOTU7 in the phylogenetic tree is highlighted by the blue box. Scale bars = 10 µM. Error bars represent standard deviations.

In order to establish whether the loss of TgOTUD3A resulted in compensatory changes in the expression levels of other TgOTU family members, we performed quantitative real-time PCR (qRT-PCR) on the wild type, the TgOTUD3A-KO mutant 2H1, and the complemented 2H1 line D5. Of the 6 OTU family members tested, 3 exhibited significant upregulation in their steady-state mRNA levels (Fig. 10A). Based on our previously published analysis, TgOTUD1B and TgOTUD1C, which are most closely related to TgOTUD3A (Fig. 10B) (34), were induced roughly 10-fold in the TgOTUD3A-KO line. Unlike TgOTUD3A, which was tightly cell cycle regulated, neither TgOTUD1B nor TgOTUD1C was cell cycle regulated (36) (http://www.toxodb.org). In contrast, of the 2 cell cycle-regulated TgOTU members (34, 36) (http://www.toxodb.org), TgOTU7 was also upregulated roughly 3-fold, while TgOTU5 was not (Fig. 10A). While these changes occurred in response to the loss of TgOTUD3A (in either clone 2H1 or 2C3), they were not restored to wild-type levels upon complementation (Fig. 10A), providing credence to the notion that a fundamental and irreversible change in the expression landscape had occurred within the KO parasites. The irreversibility of this change and the alterations in the patterns of replication resulting in the increase in the multiple-daughter phenotype could suggest a shift in the cell cycle architecture within the TgOTUD3A-KO (and complemented) parasites.

## DISCUSSION

Unlike conventional eukaryotic cell cycles, which are defined by discrete steps and are unidirectional, the replication cycles of Apicomplexa tend to possess overlapping stages that can contain phases that are repetitive and thereby no longer unidirectional (see Fig. S1 in the supplemental material) (3). Functionally, the apicomplexan cell cycle can be broadly classified into the nuclear and budding cycles. The variations in the organization of the nuclear cycle and the patterns by which it transitions into the budding cycle define the three major replication strategies (endodyogeny, schizogony, and endopolygeny), each of which has different capacities with regard to the number of progeny generated during the replicative cycle (35, 47). While endodyogeny produces 2 daughters per cycle, schizogony and endopolygeny each produce multiple progeny. *Toxoplasma* genomes encode the capacity to execute all three replication mechanisms in a life cycle stage-dependent manner. The mechanistic basis restricting the parasites to a specific mechanism in a particular life cycle stage has remained unknown. Recent studies show that the bipartite centrosome in *T. gondii* plays a major regulatory role in determining the ploidy and, by extension, the number of progeny (35, 48). Cell cycle control is mediated to a significant degree by the activity of atypical cyclins and cyclin-dependent kinase (Cdk)-related kinases (Crks), serving as checkpoints in the progression of the *T. gondii* cell cycle (44, 49).

The TgOTUD3A-KO mutant is unique in that it appears to have partially altered the regulatory landscape, which manifests as *T. gondii* tachyzoites deploying the replication strategies that are typically used in the feline gut at the onset of the sexual cycle. Despite being genetically clonal, TgOTUD3A-KO parasites can propagate by multiple replication strategies even within the same vacuole (Fig. 2 and 3). This suggests a defect that is not entirely penetrant and likely related to a not-yet-determined factor(s), the absolute levels of which define the thresholds and, thereby, the phenotypic outcomes related to the specific choice of replication strategy. As such, the differential phenotypic outcomes for the TgOTUD3A-KO parasites must be determined at the level of individual parasites and governed at a posttranslational level consistent with the dysregulation of ubiquitination/deubiquitination mechanisms to afford a highly sensitive but plastic means of control affecting cell cycle strategy and progression.

The understanding of the role of ubiquitin-mediated mechanisms in the regulation of the cell cycle of *Toxoplasma* and other Apicomplexa is in its infancy. Our recent work established that the progression of endodyogeny is associated with dynamic changes in the relative levels and spatial distribution of K48-, K11-, and K63-linked polyubiquitin chains (34). The complexity of the phenotypes associated with the TgOTUD3A-KO parasites described in the present study matches the confirmed specificity of TgOTUD3A for the K48 linkage (34), a modification associated with directing ubiquitin-mediated protein turnover (20). Notably, the levels of K48-linked polyubiquitin are highest at the S/M transition and the early phases of cytokinesis, coinciding with the transcript and protein expression peaks of TgOTUD3A expression (Fig. 1A) (34). This spatiotemporal expression profile suggests that functions of TgOTUD3A at the S/M transition and entry into cytokinesis are needed to achieve the absolute levels of the critical target(s) to allow for the progression of endodyogeny. This is entirely consistent with a threshold-driven mechanism underlying the selection of the replication strategy.

A recently completed proteomic analysis of the *Toxoplasma* (tachyzoite) ubiquitome established that 35% of ubiquitin-modified proteins are themselves transcriptionally regulated in a cell cycle-dependent manner (19). In that study, the finding that a cohort of ubiquitin ligases and deubiquitinases are transcriptionally controlled in a cell cycle-dependent manner suggests that the establishment of a dynamic balance of key factors by ubiquitin-mediated turnover is critical to ensure the fidelity of cell cycle transitions. Among ubiquitinated targets, histones, histone-modifying enzymes, DNA licensing factors, and enzymes regulating epigenetic processes point to the centrality of ubiquitin-mediated regulation in ensuring the fidelity of replication (19). A recent functional study further shows that one Crk family protein, Crk5, and its interacting partner ECR1 (essential for chromosome replication 1), regulating DNA licensing at the origin of replication, are regulated by ubiquitin-mediated mechanisms (44).

Identifying the relevant target of TgOTUD3A responsible for the observed cell cycle phenotypes is a challenging task for multiple reasons. As an exodeubiquitinase, TgOTUD3A recognizes the K48 polyubiquitin chain (34) rather than the modified target protein, with the K48 linkage representing the most abundant polyubiquitin species in the parasite (34). In addition, the loss of TgOTUD3A is compensated for by the overexpression of three related TgOTUs (TgOTUD1B, TgOTUD1C, and TgOTU7) (Fig. 10), of which only TgOTU7 is cell cycle regulated at the transcriptional level (34). This complicates the identification of the target using comparative proteomics, as one cannot distinguish whether the putative target is destabilized (by the loss of the TgOTUD3A) or stabilized (by the overexpression of one or more of the related TgOTUs).

Toward establishing the mechanistic basis for the multiple-daughter phenotype, our attention was directed at the *Toxoplasma* centrosome. Unlike the centrosome of higher eukaryotes, the *Toxoplasma* centrosome is a bipartite structure, comprised of compositionally distinct inner and outer cores (35, 50). Within this framework, the inner core of the centrosome (CEP250L1-HA was used as a marker) correlates with the control of the nuclear cycle, while the outer core (Centrin-1 was used as a marker) is linked to ensuring the fidelity of cytokinesis (35). Notably, during the nuclear cycle, when progression to cytokinesis is blocked, the outer core has limited function (35). This could be a potential regulatory mechanism to ensure the repetition of the nuclear cycle, as observed in schizogony and endopolygeny. Full outer core functionality is restored during the final nuclear cycle and upon entry into the budding cycle, when DNA synthesis proceeds to mitosis/karyokinesis and on to cytokinesis with the formation of multiple daughter buds (35). In *Toxoplasma* tachyzoite endodyogeny, the norm is a single nuclear cycle that is immediately coupled with an overlapping budding cycle (5). This restriction is partially lost in the TgOTUD3A-KO parasites, as we clearly see the uncoupling of the nuclear and budding cycles (within a subset of parasites), either because of extended DNA replication (beyond 2N), resulting in a large polyploid nuclear mass (3N or 4N or more) (endopolygeny-like), or reinitiation of DNA replication after the first round of karyokinesis without entering into the budding cycle (schizogony-like) (Fig. 2 and 6). The synchronized entry into the budding cycle, regardless of the number of daughter parasites within individual parasites and within a vacuole (Fig. 2B to D), suggests that the defect associated with the multiple-daughter phenotype precedes cytokinesis and is corrected prior to the initiation of the budding cycle. This transient defect has been determined to be a high frequency of aberrant centrosome duplication following the first duplication. These deviations manifest in two different ways. One is an imbalanced reduplication (1 of the 2 centrosomes reduplicating) that results in progression to a 3N or greater odd-number ploidy. Given that the centromeres of the chromosomes are sequestered to the centrocone throughout the cell cycle (51), this imbalanced reduplication happens in two different formats, either after the first round of karyokinesis is complete (schizogony-like) (Fig. 7A and D) or before the karyokinesis occurs (endopolygeny-like) (Fig. 7B and C). In the latter case, even though the karyokinesis is not complete, presumably the mitosis is complete in the polyploid mass, as described for *Sarcocystis* endopolygeny (52), and most likely, differential signaling events through one of the centrosomes causes this aberrant reduplication. The orphan centrosome resulting from this imbalanced reduplication, which is lacking the partner centrocone (Fig. 6D, MORN1), indicates that the connection to the centrosome cannot have been established, probably because the synthesis of the third genome equivalent was underway and it was therefore not ready for mitosis. The other deviation from the norm is the balanced reduplication wherein both the centrosomes reduplicate either before or after the first round of karyokinesis is complete and that can be explained by signaling events similar to those described above. Of interest is a recent finding of a role for the ECR1/TgCrk complex in centrocone duplication and the ubiquitin-mediated turnover of ECR1, which is related to the geminins in higher eukaryotes (44). Geminins play a critical role in preventing relicensing of DNA synthesis, chromosome duplication, and spindle formation (53, 54). It is possible that a geminin-like molecule could be a target of TgOTUD3A in regulating the centrosome duplication and maintaining the fidelity of replication.

The drivers of aberrant reduplication may be related to the degree of centrosome maturity following the initial duplication, as suggested by differences in the intensities of the centrosome signals (size) within individual parasites (Fig. 4 and 5). It is unclear, however, whether aberrant reduplication is exhibited by the mature mother or putatively immature daughter centrosome, as specific markers for centrosome maturation are not known. Of note, however, a study examining the role of centriolar plaques in asymmetrical mitotic division (1 nucleus divides while another does not) in *Plasmodium* suggests that it is the mother centrosome which initiates reduplication in the second S phase (11).

One obvious question now arises, as to how a cytoplasmic factor like TgOTUD3A (34) can regulate the nuclear cycle by controlling centrosome duplication, as all Apicomplexa, including *T. gondii*, undergo closed mitosis, wherein the nuclear envelope does not completely break down. Evidence from the fission yeast, *Schizosaccharomyces pombe*, which also undergoes closed mitosis, reveals that there is a transient localized breakdown of the nuclear envelope that allows localized contact between the cytoplasm and nucleoplasm surrounding the area where the centrosome is tethered to the nuclear envelope (55). Such a function may be associated with centrocone dynamics (44, 51) or could provide an alternative mechanism to engage the spindle checkpoint, arresting further DNA synthesis for the specific genome complement associated with a given centrosome. There is ample evidence from literature on mammals that ubiquitination controls the duplication dynamics of centrosomes (56–58). The overexpression of a cell cycle-dependent E2 Ub ligase (which is functionally equivalent to the knockdown or knockout of a cell cycle-regulated DUB), UbcH10, results in the generation of multiple centrosomes, which despite being in clonal lines, occurs in 28 to 33% of the cells (56), a very similar phenotype to that observed in TgOTUD3A-KO parasites.

The multiple-daughter and/or aberrant endodyogeny phenotypes have been reported previously in other *T. gondii* mutants with mutations affecting lipid export (NPC) (59) and protein sorting through the Golgi complex (Rab6) (60). Although these mutants are viable (at least in the short term for the Rab6 mutant [60]), none of these studies dissected these phenotypes from the perspective of the cell cycle and centrosome dynamics. Interestingly, the nuclear phenotypes in those mutant parasites are found to be similar to that of TgOTUD3A-KO parasites, suggesting that perturbation of the functions of these genes impacts the cell by mediating the dysregulation of cell cycle checkpoints that govern which replication strategy to adopt. In contrast, most of the conditional (*ts*) mutant parasites that uncouple the nuclear and budding cycles produce multiple progeny that are not viable (41). However, the TgOTUD3A-KO mutants, which appear to transiently uncouple the nuclear and budding cycles, are viable, as shown by plaque assay, competition assay (Fig. 1C and D), and morphology (Fig. 2 and 3). The facts that this multiple-daughter phenotype is not fully penetrant and is not reversed by complementation (Fig. 8) suggest some fundamental changes in the physiological landscape, defining what is now a new normal in both the mutant and complemented lines (Fig. 8). The absence of credible off-target mutations (Fig. 9) predicts compensation by other factors. Such compensatory changes may be mediated by the upregulation of other closely related TgOTUs (Fig. 10), allowing adaptation to this new normal state.

Interestingly, the *Plasmodium* ortholog (PF3D7_0923100) of TgOTUD3A is only expressed at the schizont stages in a genome-wide microarray analysis (PlasmoDB) (61). In addition, an anti-TgOTUD3A antibody cross-reacts with the orthologous protein only from purified schizonts and not from other erythrocytic stages (data not shown), suggesting potentially conserved cell cycle-specific roles in Apicomplexa, governing the transition from the nuclear to the budding cycle.

In summary, the analysis of the TgOTUD3A-KO mutant reinforces a role for ubiquitination-mediated mechanisms in the sophisticated decisions underlying the progression and organization of the apicomplexan cell cycle. It further demonstrates that ubiquitin-mediated mechanisms likely control key switches governing the mechanisms that typically restrict the selection of cell cycle architecture of *Toxoplasma* in a life cycle-determined manner. By loosening the controls that restrict tachyzoites to endodyogeny, a subset of TgOTUD3A-KO parasites follow cell cycle progression strategies while retaining the capacity to undergo endodyogeny. The basis of this plasticity appears to be linked to the centrosome dynamics, which reinforces earlier studies (35). The analysis further presents a potential mechanistic foundation to dissect all three replication strategies displayed by Apicomplexa. These insights reveal how *Toxoplasma* executes three distinct replication strategies across its life cycle, ensuring the fidelity of cell cycle progression potentially tuned to the requirements of the specific life cycle stage.

## MATERIALS AND METHODS

### Parasite culture and maintenance.

Type I RHΔHXGPRT and RHΔHXGPRTΔku80, HA-tagged TgOTUD3A and TgOTUD3A-KO, and complemented *Toxoplasma gondii* lines were cultured in primary human foreskin fibroblast (HFF; ATCC) cells in Minimal Essential Medium with alpha modification (α-MEM) supplemented with 7% heat-inactivated fetal bovine serum (FBS; Gemini Bio-Products, West Sacramento, CA), 100 U/ml penicillin, 100 mg/ml streptomycin, and 2 mM l-glutamine (Gibco BRL, Rockville, MD) at 37°C and 5% CO_2_ in a humidified incubator. Modified parasite lines selected on the basis of drug resistance were grown on medium containing appropriate drugs.

### Epitope tagging of TgOTUD3A.

The TgOTUD3A gene (TGGT1_258780) was tagged at the C terminus with a triple-HA tag by using the pHA_3x_LIC tagging plasmid (kind gift from Peter Bradley, UCLA) as described elsewhere (34). Both the wild-type and TgOTUD3A-KO lines were tagged using a fosmid containing a tagged copy of CEP250L1-HA and the chloramphenicol resistance gene (kind gift from Michael White, USF), and clonal lines were isolated for further analysis.

### TgOTUD3A knockout and complementation by CRISPR-Cas9 gene editing.

The TgOTUD3A gene open reading frame was disrupted by using clustered regularly interspaced short palindromic repeat (CRISPR)–CRISPR-associated gene 9 (Cas9)-mediated gene editing technology (62). The base CRISPR-Cas9 plasmid (37) was a kind gift from L. D. Sibley, Washington University, St. Louis, MO. TgOTUD3A gene (TGGT1_258780)-specific guide RNAs (gRNAs) were designed using the online E-CRISP portal (http://www.e-crisp.org/E-CRISP/designcrispr.html). Based on the ranking, 4 gRNA sequences were selected to replace the endogenously encoded anti-TgUPRT gRNA, using the Q5 mutagenesis kit (New England BioLogicals) as previously described (37). For selection based on pyrimethamine resistance, a mutated version of the dihydrofolate reductase (DHFR) cassette (63) was amplified from the pBioID-HA_3x_-DHFR plasmid (Peter Bradley, UCLA) by using a primer pair attached to the 20-nt sequence flanking the CRISPR target sequence. All the primers used to make the TgOTUD3A gene knockout mutant by CRISPR are listed in Table S1 in the supplemental material. Both the PCR-amplified DHFR expression cassette and the CRISPR-Cas9 plasmid containing TgOTUD3A-specific gRNA were transfected into *Toxoplasma gondii* strain RH. At 24 h posttransfection, the parasites were harvested and syringe passaged. The syringe-passaged material was filtered using membrane filters (CellTrics; Partec, GmbH) with a 50-micron cutoff prior to sorting for Cas9-green fluorescent protein (GFP) on a flow cytometer (Sony SY3200, installed in a biosafety level II cabinet). Sorted parasites were used to infect HFF cells in a 6-well plate. Following 2 passages in the pyrimethamine-containing medium, the parasites were cloned by limiting dilution. Each line was grown, and the parasite lysates were tested for immunoreactivity by Western blot analysis using the mouse polyclonal anti-TgOTUD3A antibody (34). Genomic DNA (gDNA) was isolated from the lines that showed no TgOTUD3A protein expression. The integration of the DHFR cassette into the double-stranded DNA break site was confirmed by PCR, using the gDNA as the template. The PCR products were sequenced to confirm that the knock-in mutation disrupted the open reading frame and thereby caused the loss of protein expression.

10.1128/mBio.01846-17.9 TABLE S1  List of primer sets used in the study, arranged by specific application. Download TABLE S1, PDF file, 0.1 MB. Copyright © 2017 Dhara et al. 2017 Dhara et al. This content is distributed under the terms of the Creative Commons Attribution 4.0 International license.

Complementation was done by using the same CRISPR-mediated gene-editing approach. Two CRISPR gRNA-containing plasmids targeting the flanking region of the DHFR cassette in the TgOTUD3A locus, along with an HA-tagged copy of functional full-length TgOTUD3A DNA (with its endogenous promoter), were transfected into the TgOTUD3A-KO 2H1 line, and clones were selected based on their resistance or sensitivity to mycophenolic acid (MPA)/xanthine and pyrimethamine. The expression of the complemented TgOTUD3A gene was tested by immunofluorescence and immunoblot analyses and further verified by whole-genome sequencing.

### Plaque assay.

HFF monolayers were infected with a small number (150 parasites/well in 6-well plates) of parasites of the wild-type RH, TgOTUD3A-HA, and TgOTUD3A-KO lines. The plaque sizes were monitored on a daily basis. On day 6 postinfection, the wells were washed with 1× phosphate-buffered saline (PBS), fixed with ice-cold methanol (MeOH), and stained with crystal violet for visualization of the plaques. Images of a total of 150 plaques were randomly acquired (using a 10× objective) from all the parasite lines from three independent experiments. The areas of the plaques in arbitrary units were determined by measuring the plaques using NIH ImageJ software.

### Competition assay.

The TgOTUD3A-HA (wild-type) and TgOTUD3A-KO lines were used for a head-to-head competition assay. Based on the sequence variation at the TgOTUD3A locus due to the introduction of the HA-DHFR drug cassette at the 3′ UTR in the wild-type HA-tagged line and the DHFR drug cassette near the ATG start codon, we designed specific primer sets that will only amplify the parasite line-specific genomic DNA. The assay was standardized and validated by mixing different ratios of both of the genomic DNAs and using a single primer set for both combinations. The real-time data (not shown) for standardization faithfully represented the real combinatorial ratios used for specific reaction mixtures. For the actual experiment, HFF monolayers in each well of a 6-well plate were infected with equal numbers (2 × 10^5^ and 5 × 10^3^ for 24-h and 48-h time points, respectively) of each parasite line. Parasites were harvested after 24 and 48 h postinfection with equal numbers of wild-type (TgOTUD3A-HA) and TgOTUD3A-KO parasites, and total gDNA was collected and treated with RNase to remove RNA contamination. In each real-time PCR, 100 ng of genomic DNA was used, along with a specific set of primers (either wild type or TgOTUD3A-KO specific). The amount of template was normalized using TgSAG1 expression. Real-time PCR data were obtained using a Bio-Rad CFX instrument and analyzed by the cycle threshold (ΔΔ*C*_*T*_) method.

### Cell cycle (DNA content) analysis.

To measure the parasite DNA content, a previously described protocol (64) was followed. In brief, HFF monolayers were infected with both WT and TgOTUD3A-KO parasites. At 24 h and 36 h postinfection, parasites were harvested, syringe passaged, and filtered through 10-μm filters (CellTrics; Partec, GmbH). For a negative control, host cells only were harvested and treated the same way. Parasites fixed with filtered ethyl alcohol (EtOH; overnight fixation at −20°C) were treated with RNase, stained with the DNA dye SYTOX green (Invitrogen), and analyzed with a flow cytometer (Sony SY3200) by collecting 50,000 events for each parasite line. Data were analyzed using WinList 8.0 (Verity Software House).

### Immunofluorescence assay.

HFF monolayers were grown in confluent monolayers on coverslips in 24-well plates and infected (1 × 10^4^ per well) with either wild-type (RH) or TgOTUD3A-KO parasites. Around 16 to 24 h postinfection, infected cell monolayers were fixed as previously described (65). The following primary antibodies were used at the indicated dilutions: rabbit anti-HA antibodies (from Covance, 1:250, and from Cell Signaling, 1:100), rat anti-TgIMC3 antibody (1:1,000) (66), mouse anti-TgIMC1 antibody (1:1,000) (kind gift from Gary Ward, University of Vermont), rat anti-MORN1 antibody (1:10) (43) (kind gift from Ke Hu, Indiana University), rabbit anti-SAG1 antibody (1:30,000) (kind gift from John Boothryod, Stanford University), mouse anti-*Chlamydomonas* centrin monoclonal antibody (20H5) (EMD Millipore), mouse anti-TgAtrx1 antibody (apicoplast; 1:1,000) (67), mouse anti-TgMIC3 antibody (microneme; 1:1,000) (68), mouse anti-TgROP7 antibody (rhoptry, 1:1,000) (69), mouse anti-TgF1β antibody (mitochondrion; 1:1,000) (65) (gift from Peter Bradley), mouse anti-TgGRA3 antibody (parasitophorous vacuole membrane; 1:1,000) (70), and rabbit anti-TgSAG1 antibody (surface antigen; 1:3,000) (71). Slides were visualized using a Zeiss AxioVision stand with a 100× 1.4 numeric aperture (NA) oil immersion objective and images acquired using a high-resolution grayscale Zeiss AxioCam MRM digital camera. Where relevant, Z stack images were acquired and were deconvoluted using an iterative algorithm (AxioVision Deconvolution Suite; Zeiss). Grayscale images were pseudocolored to reflect the emission of the specific fluorophore, brightness and contrast were adjusted, and the merged images were generated using Adobe Photoshop CS6. All manipulations (brightness/contrast) of the images acquired were applied uniformly to the images presented.

### qRT-PCR assay.

Total RNA was extracted from 1 × 10^8^ RH and TgOTUD3A-KO parasites using RNeasy columns (Qiagen) in which on-column DNase digestion was done to degrade all the genomic DNA. Total RNA was quantified on a NanoDrop 1000 instrument (Thermo Scientific). Equal amounts (maximum 1 µg) of total RNA were used for cDNA synthesis (Promega reverse transcription system). Briefly, PCRs using 20 µl each of cDNA synthesis reaction mixture [4 µl MgCl_2_ (25 mM), 2 µl 10× reverse transcriptase buffer, 2 µl deoxynucleoside triphosphate (dNTP) mixture, 0.5 µg oligo(dT) primer, 0.5 µl RNase inhibitor, and 1 µl AMV reverse transcriptase] were run under the following PCR cycling conditions: 42°C for 60 min, 95°C for 5 min, and 4°C for 5 min. For each TgOTU, a primer pair capable of amplifying a 200-bp PCR product was designed (see Table S1 for primer sequences) and as a housekeeping internal control, a SAG1 (TGGT1_233460) primer pair was used to amplify the SAG1 transcript to normalize the initial transcript copy number. Primers that only amplified products of the expected size (single band on agarose gel) were used for this assay. The quantitative real-time PCR (qRT-PCR) mixture (10 µl), containing iTaq Universal SYBR green supermix (BioRad), template cDNA (100 ng/reaction mixture volume), and specific primer pairs, was run on a CFX Connect real-time PCR detection system (Bio-Rad) for 40 cycles under the following conditions: amplification reaction at 95°C for 3 min, 95°C for 15 s, and 60°C for 30 s, and melt curve analysis from 65°C to 95°C in a 0.5°C increment every 5 s. Real-time PCR data were analyzed by the ΔΔ*C*_*T*_ method.

### Statistical analysis.

Based on the data types and distribution, one-way analysis of variance (ANOVA) tests were done to determine the levels of significance for the mean differences between wild-type and TgOTUD3A-KO parasites for a number of variable traits that were looked at, such as plaque size, parasite number per vacuole, number of daughter scaffolds per gravid parasite, etc. The *P* value was determined by the Brown-Forsythe test and corrected Bartlett’s test (corrected for any deviation from Gaussian distribution). The difference between any two group means (for the specific test metric) was analyzed using Tukey’s multiple-comparison test with a 95% confidence interval. The details of each test metric and level of significance are mentioned in Results and in individual figure legends GraphPad Prism version 6.0 (La Jolla, CA) was used to generate all the bar graphs and to perform all statistical analysis.

### Next-generation whole-genome sequencing and analysis.

Genomic DNA was isolated from 4 clonal lines (WT, KO mutant 2C3, KO mutant 2H1, and Compl-2H1 [D5]), and sequencing libraries were made using the TruSeq DNA PCR-free method, run on lanes of split flow cells, and sequenced by Illumina HiSeq 2500 rapid run sequencing at the University of Kentucky Genomics Core Laboratory. The output paired-end 100- to 125-nucleotide reads were mapped against the RH reference genome. Mapped reads were analyzed through a pipeline of programs/tools to find unique and shared mutations. A detailed description of the analysis is available in Text S1.
