# Supplementary figures and images for: Ablation of an Ovarian Tumor Family Deubiquitinase Exposes the Underlying Regulation Governing the Plasticity of Cell Cycle Progression in Toxoplasma gondii
Source: mBio. 2017 Nov 21;8(6):e01846-17. doi: 10.1128/mBio.01846-17 (PMC5698556; doi:10.1128/mBio.01846-17)

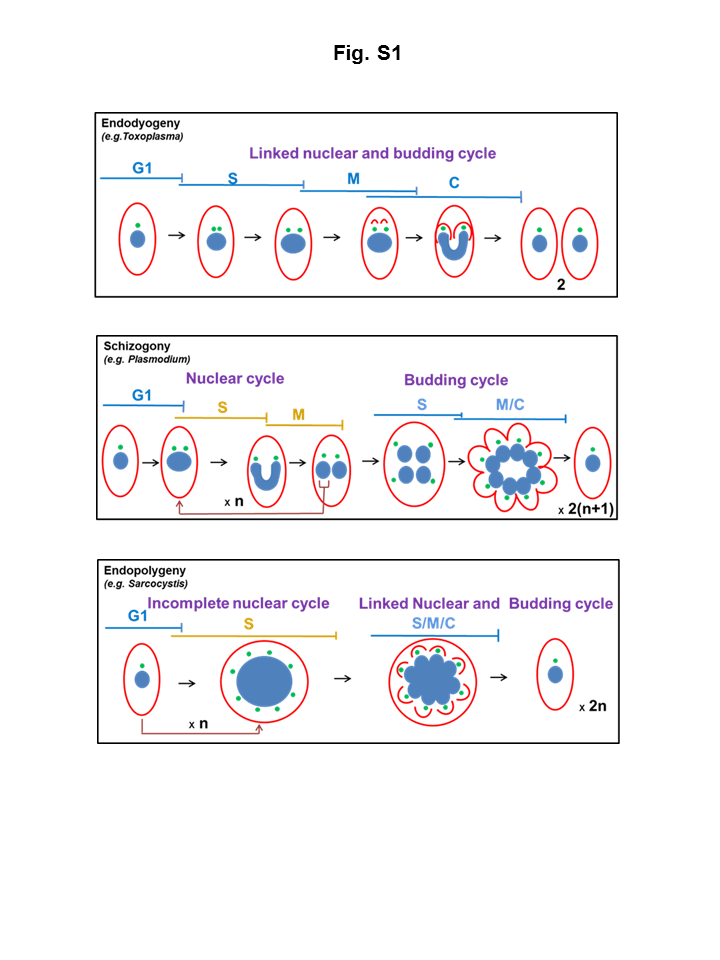

Supplement: FIG S1 [file mbo006173593sf1.tif]

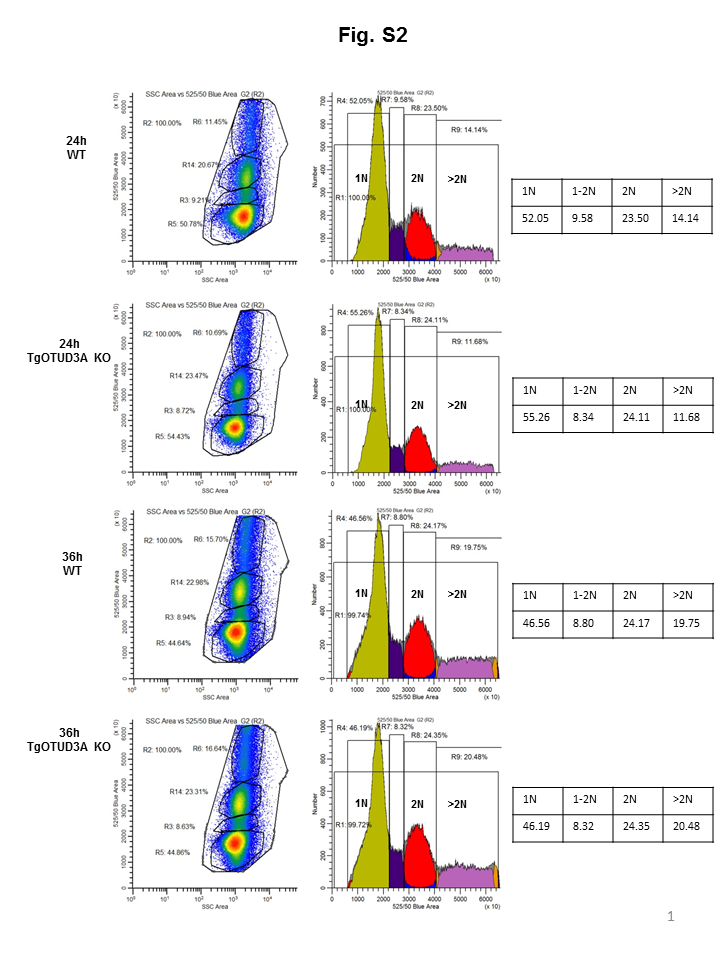

Supplement: FIG S2 [file mbo006173593sf2.tif]

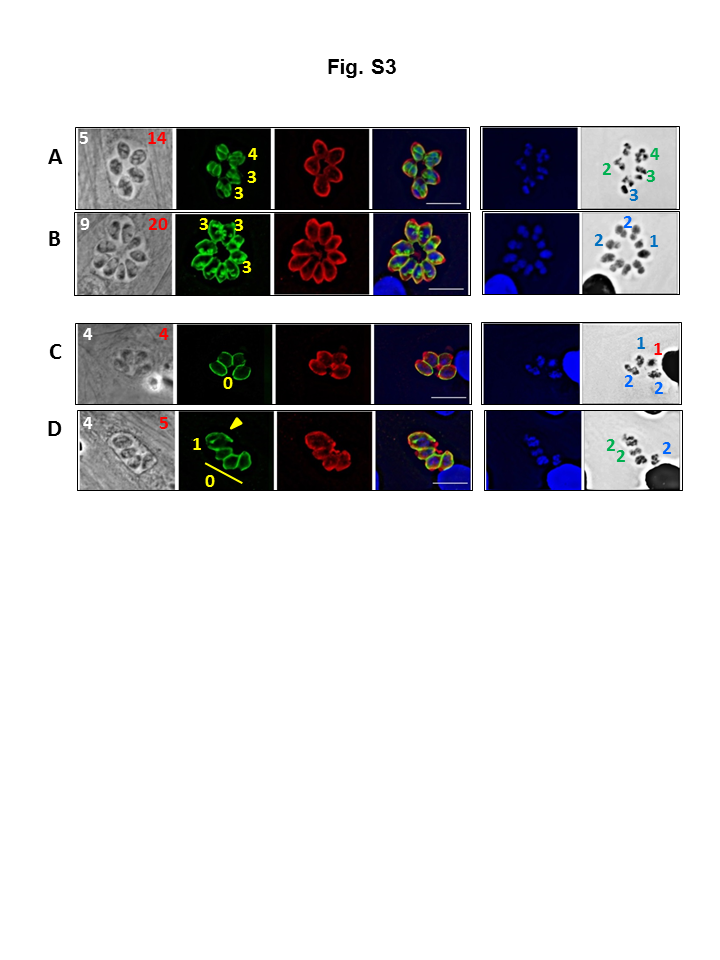

Supplement: FIG S3 [file mbo006173593sf3.tif]

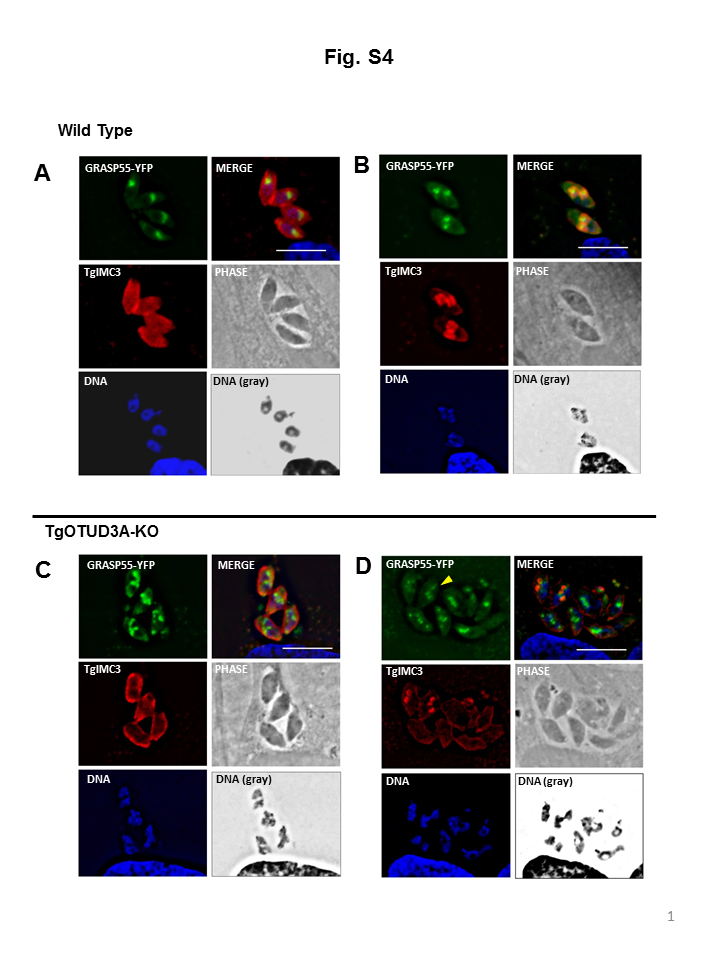

Supplement: FIG S4 [file mbo006173593sf4.tif]

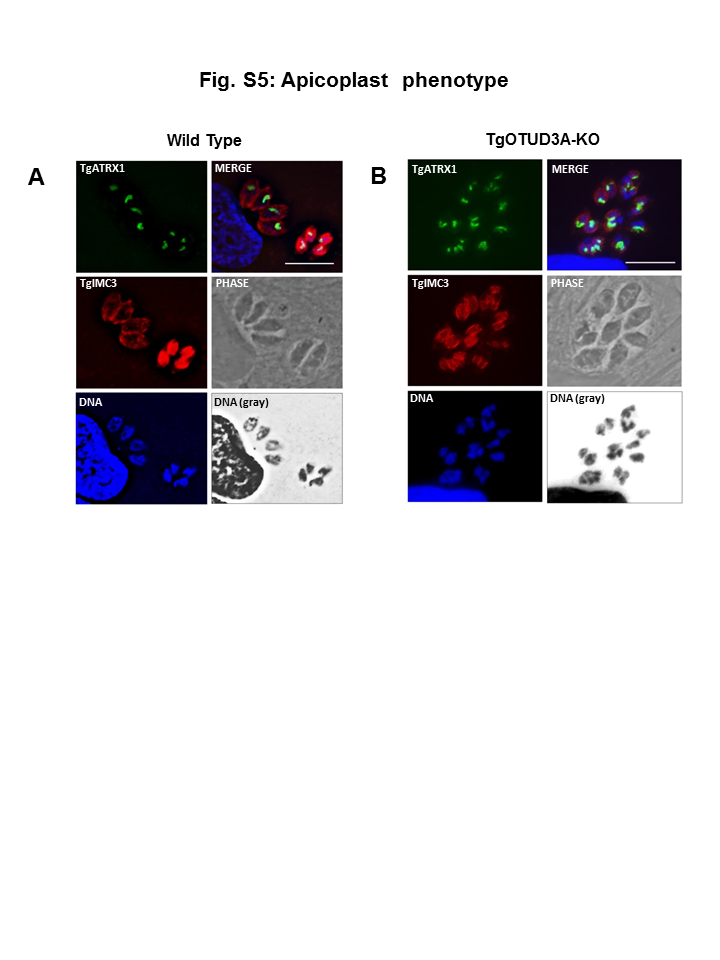

Supplement: FIG S5 [file mbo006173593sf5.tif]

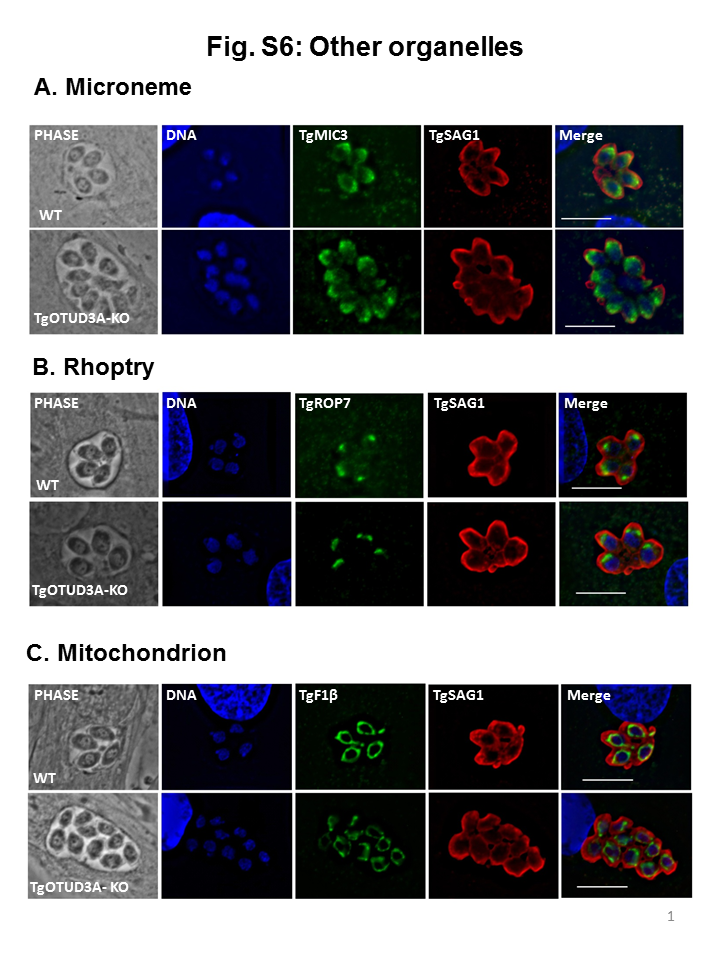

Supplement: FIG S6 [file mbo006173593sf6.tif]

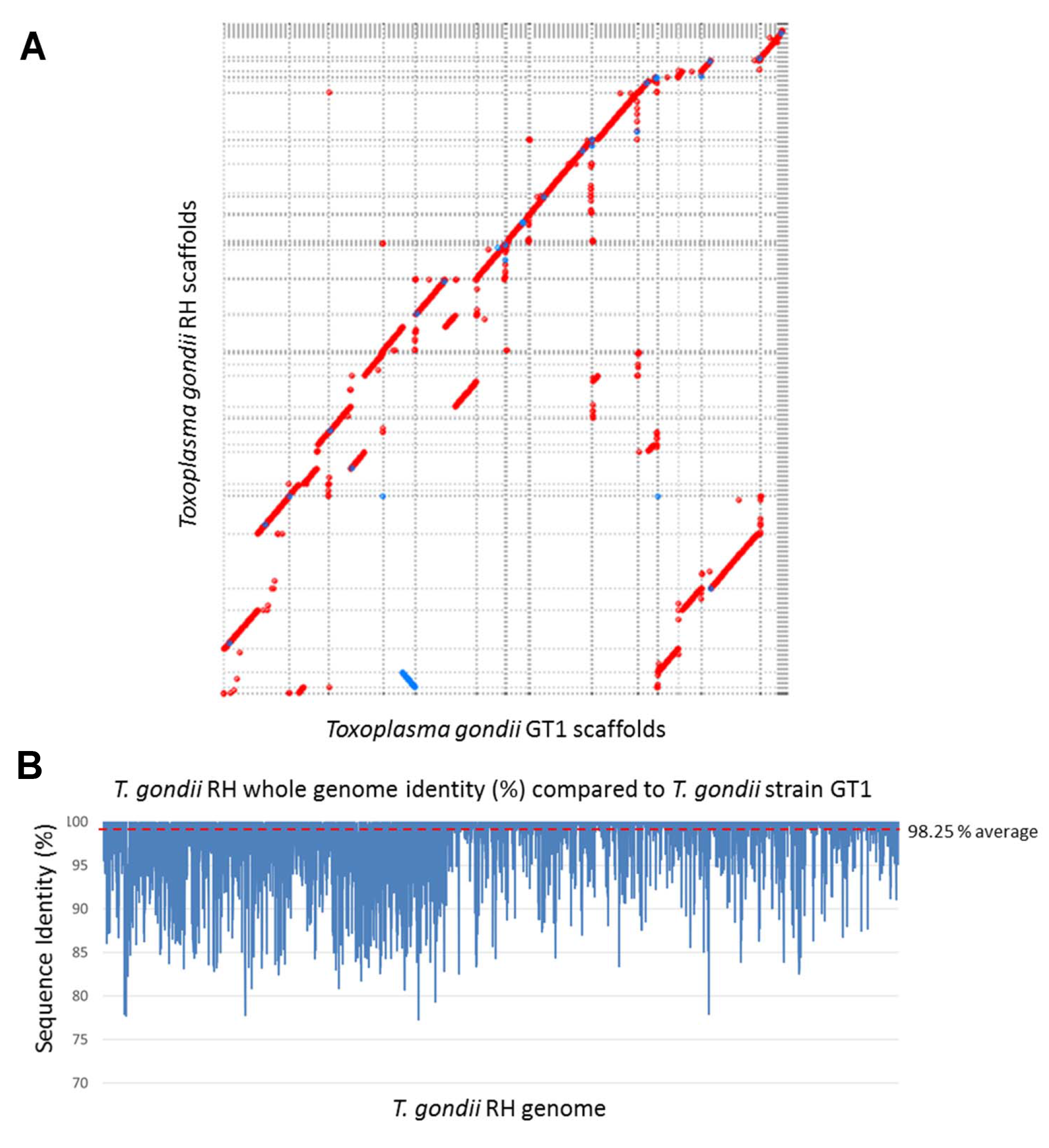

Supplement: FIG S7 [file mbo006173593sf7.tif]
