# Supplementary material for: Ablation of an Ovarian Tumor Family Deubiquitinase Exposes the Underlying Regulation Governing the Plasticity of Cell Cycle Progression in Toxoplasma gondii
Source: mBio. 2017 Nov 21;8(6):e01846-17. doi: 10.1128/mBio.01846-17 (PMC5698556; doi:10.1128/mBio.01846-17)
Supplement: TEXT S1 [file mbo006173593s1.pdf]

Text S1 A

Whole genome sequencing analysis

We used a recently published whole genome sequence data for the Type I RH strain [1] as the reference to perform all analyses as the parental (WT), mutant and complemented lines were in this genetic background. No annotation is currently available for this RH genome [1]. We therefore aligned the RH genome [1] and the annotated Type I GT1 genome (www.ToxoDB.org) using the MumMer aligner tool [2] with an identity threshold set at 70%. The two genomes were found to be 98.25% identical (Fig. S8). Based on the identity concordance between both genomes we set the best parameters to perform an annotation transfer between both genomes using the rapid annotation transfer tool (RATT) [3] and created a homologous draft annotation gff file to perform all our analysis based on the Type I GT1 annotation information from ToxoDB. A flow chart representing the workflow for the analysis of the NGS data is presented in Figure 1.

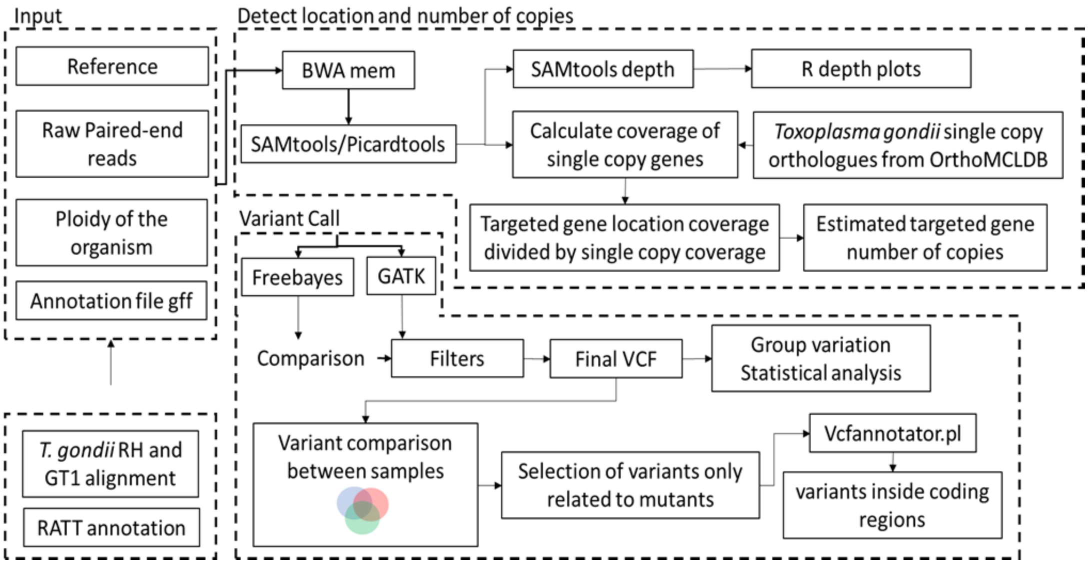

Figure 1: Workflow of the whole genome sequencing analysis

i. Read Depth Coverage analysis

The raw paired-end short 100-125 nt reads obtained by illumina Hiseq2500 from four samples (one WT, two KOs (2C3, 2H1), and one complemented line (Comp-D5) were aligned to the GT1 alignment based annotated RH reference assembly with the ploidy set to one. In the first step of the analysis, raw reads were run through BWA mem [2] , SAMtools package [4] and PicardTools (http://broadinstitute.github.io/picard). The parsed output from these analyses, a sorted binary alignment file (BAM), was then submitted to SAMtools depth script [4] to recover the read depth coverage (RDC) of each base of these genomes. Using an in-house shell script, we calculated the average depth coverage of each of the four genomes we sequenced, and validated it by calculating the average depth coverage of some known single copy genes that were randomly picked from OrthoMCL-DB [5].

Copy number analysis of DHFR and HXGPRT gene

After obtaining the “single copy” average coverage for each of the four genomes, we selected the target loci for DHFR/TS (TGGT1_249180: chromosome XII between positions 4,294,324 and 4,300,363) and HXGPRT (TGGT1_200320: chromosome VIII between 6,773,060 and 6,774,877) genes to determine if average coverage of the reads in those regions reflects any increase due to insertion of drug resistance cassettes as knock-in repair patch for making KO (DHFR/TS) or complemented line (HXGPRT) using CRISPR/Cas9.

ii. Flanking sequence analysis for DHFR/TS gene copies

As the copy number analysis revealed that more than one copy of the DHFR cassette got integrated in the two independent KO genomes, we further investigated if the extra

copies of DHFR/TS^R were inserted tandem or integrated elsewhere at the off-target locations. To analyze that we took 50 nt flanking sequences from both ends of DHFR cassette (5' and 3' UTR of DHFR cassette respectively) and performed an alignment analysis to the raw reads using Burrows Wheeler Aligner [2] to identify the loci of insertions. The coverage visualization plot was made using R plot script [6]. This analysis revealed that the DHFR/TS drug selection cassette integrated in either a single or double copy exclusively in the TgOTUD3A locus.

iii. Variant call analysis

Mutations other than random insertion of drug cassettes caused by our CRISPR approach are mainly of two kinds - single nucleotide polymorphism (SNPs) and insertions or deletions (InDELs). So we investigated if the CRISPR has caused any of such off-target mutations which might explain the failure of the addition of the TgOTUD3A locus to restore the wild type phenotypes in the complemented line, by performing a global variant call analysis. We used the genome analysis toolkit (GATK) unifield-genotyper [7] to classify variants by two quality control steps, using mapping quality (MQ > 25), minimum depth (DP > 5), sequence quality (Q > 30) and quality depth (QD > 1.5). The output lists of variants from GATK were further verified using another variant call analysis method, Freebayes [8] at the default setting and using ploidy equals to one. The variant calls for each sample were filtered and the final variant call files (VCFs) were further analyzed to see how many of them were unique or shared between any of the four genomes we sequenced. The result of this analysis was plotted in the Venn diagram V1.6.17 R package (https://www.rdocumentation.org/packages/VennDiagram/versions/1.6.17). To evaluate

the significance of variants, whether random or resulting from off target effects of CRISPR-Cas9, we performed a Poisson distribution test and a single factor analysis of variance (ANOVA). The Poisson test determines the randomness of the mutation between samples and the two way ANOVA detected difference between and within the samples. Finally, our interest to fish out the variants due to off-target mutations by CRISPR led us look at the shared mutations that are present in two KO lines (2H1 and 2C3) and one complemented (D5) line but absent in WT. Using the perl script vcfannotator.pl (Broad Institute, Cambridge, MA) tool and information from the annotation gff file, we identified variants that are within predicted coding sequence and in intergenic regions.

References:

1. Lau YL, Lee WC, Gudimella R, Zhang G, Ching XT, et al. (2016) Deciphering the Draft Genome of Toxoplasma gondii RH Strain. PLoS One 11: e0157901.
2. Li H, Durbin R (2009) Fast and accurate short read alignment with Burrows-Wheeler transform. Bioinformatics 25: 1754-1760.
3. Otto TD, Dillon GP, Degrave WS, Berriman M (2011) RATT: Rapid Annotation Transfer Tool. Nucleic Acids Res 39: e57.
4. Li H, Handsaker B, Wysoker A, Fennell T, Ruan J, et al. (2009) The Sequence Alignment/Map format and SAMtools. Bioinformatics 25: 2078-2079.
5. Chen F, Mackey AJ, Stoeckert CJ, Jr., Roos DS (2006) OrthoMCL-DB: querying a comprehensive multi-species collection of ortholog groups. Nucleic Acids Res 34: D363-368.
6. Team RDC (2008) R: A language and environment for statistical computing. R Foundation for Statistical Computing, Vienna, Austria ISBN 3-900051-07-0.
7. McKenna A, Hanna M, Banks E, Sivachenko A, Cibulskis K, et al. (2010) The Genome Analysis Toolkit: a MapReduce framework for analyzing next-generation DNA sequencing data. Genome Res 20: 1297-1303.
8. Garrison E, Marth, G. (2012) Haplotype-based variant detection from short-read sequencing. arXiv:12073907[q-BioGN].

Text S1 B

Anova: Single Factor

SUMMARY

Groups	*Count*	*Sum*	*Average*	*Variance*
Column 1	195	6346	32.54359	4885.352
Column 2	192	6122	31.88542	4669.359
Column 3	189	6271	33.17989	4885.467
Column 4	192	6318	32.90625	4941.625

ANOVA

Source of Variation	*SS*	*df*	*MS*	*F*	*P-value*	*F crit*
Between Groups	179.6939	3	59.89798	0.012362	0.99812	2.616558
Within Groups	3701924	764	4845.45			
Total	3702104	767				

Conclusion: F < F crit, so we do not reject the null hypothesis, what means that between all population analysied they are overall equal.

SNPs per contig

WT	2C3	2H1	Comp
197	214	211	202
2	2	3	2
10	10	10	10
1	4	3	3
4	1	1	1
1	1	1	1
1	1	1	2
2	1	1	1
1	1	1	1
1	1	1	1
2	1	1	9
1	1	8	11
1	8	9	402
8	2	397	21
7	6	22	23
383	394	26	121
23	22	115	4
27	27	4	1
116	114	1	1
4	3	1	4
1	1	2	5
1	1	4	29
3	4	32	122
2	3	124	235
32	29	235	4
118	116	4	1
231	219	1	2
4	4	3	2
1	2	2	2
1	3	3	1
3	2	3	1
3	1	1	4
2	1	4	33
2	4	29	1
2	24	1	2
4	2	2	3
32	3	2	7
1	8	12	1
3	2	1	1
3	1	2	1
9	12	3	12
1	2	12	2
1	1	2	2
1	23	1	23
1	24	23	22
12	10	19	10
2	1	9	3

1	3	3	3
22	4	3	9
21	7	9	1
9	1	1	9
3	9	11	3
3	1	1	66
9	3	3	5
1	66	70	1
9	3	5	1
3	1	1	2
69	4	1	3
5	2	3	1
1	1	3	6
2	1	1	2
2	6	7	348
1	2	1	4
6	330	354	15
1	5	4	10
358	16	14	9
1	10	10	51
4	13	9	5
16	55	43	1
9	6	2	7
12	1	1	1
52	7	7	5
4	1	1	14
1	5	5	13
5	14	14	19
1	10	13	29
6	20	23	1
16	25	26	38
10	3	3	25
24	36	33	9
30	24	28	92
3	8	7	1
31	101	94	1
24	1	2	1
7	1	1	2
97	1	1	1
1	1	2	1
1	1	8	7
1	7	1	1
2	18	17	16
9	3	1	1
1	7	9	5
19	5	4	6
2	16	13	13
6	7	10	9
6	3	3	5
17	12	9	6
9	7	9	7

4	30	33	32
8	5	6	5
7	2	2	1
34	39	48	44
4	2	2	2
2	7	8	9
39	1	1	1
2	2	2	2
10	4	3	3
1	4	8	5
2	8	11	10
3	12	13	12
4	18	14	13
10	19	21	25
14	36	42	44
16	2	2	2
27	1	3	1
50	2	7	2
2	12	140	10
2	151	52	138
11	54	2	58
150	2	19	2
57	10	3	11
2	6	23	3
9	20	38	20
6	34	32	41
25	27	311	29
41	323	307	328
31	292	9	315
342	9	13	11
314	12	8	14
9	12	32	11
11	34	254	35
7	263	2	264
32	6	6	5
277	13	16	13
6	2	4	4
10	1	1	1
4	14	14	15
1	27	37	41
14	80	85	81
35	119	125	130
85	2	1	2
122	1	1	2
2	1	1	1
1	22	19	1
1	6	6	23
1	19	26	7
21	12	16	24
9	48	52	17
22	69	73	58

	16	2	2	75
	54	2	1	1
	83	1	2	2
	2	2	7	1
	1	5	17	1
	2	12	11	6
	6	10	138	15
	18	137	51	14
	9	50	241	132
	141	234	2	54
	55	1	7	247
	238	1	17	1
	1	2	24	11
	2	6	277	12
	10	15	2	23
	17	31	75	268
	22	283	123	1
	283	1	209	70
	2	74	9	121
	71	128	2	215
	131	202	2	9
	198	10	12	2
	10	2	99	2
	2	1	180	10
	2	10	250	101
	10	95	4	180
	101	176	3	259
	182	246	1	1
	242	2	3	4
	2	3	49	2
	3	1	192	1
	2	1	2	4
	1	4	10	51
	3	51	6	198
	50	169	10	2
	203	2	7	10
	2	7	1	9
	7	7	1	7
	9	7	7	7
	12	8	4	1
	7	1		2
	1	8		7
	1	4		8
	1			
	7			
	4			
Total	**6346**	**6122**	**6271**	**6318**

Text S1 D

Ref Gene ID	Description	Scaffold Position	Ref (RH wild type)	Alt	Variation	Changes to Aa or Protein [Stop / Full length Aa]
TGGT1_290190	Hypothetical Protein	1489551	C	CT	Insertion	[82/1241]
TGGT1_290180	AP2IX-6	1489551	C	CT	Insertion	[82/1241]
TGGT1_292170	ve histone lysine methyltransferase	169972	G	GCTCCAC	Insertion	626Ser-Leu-His-627Gly
TGGT1_295710	omain, G-beta repeat-containing p	1421420	CTTCCTCG	C	Deletion	[3564aa/ 3641]
TGGT1_215090	Putative ATP binding protein	3009173	C	T	SNP	769TCC/TCT (Ser)
TGGT1_250500	Hyppothetical Protein	5711	T	G	SNP	206GAG (Glu) / GCG (Ala)
TGGT1_305890	Hyppothetical Protein	721086	G	A	SNP	1103GAG/GAA (Glu)
TGGT1_264420	Lipoprotein	78	T	A	SNP	22TGG (Trp) / AGG (Arg)
TGGT1_264420		64	G	A	SNP	17GGG (Gly) /GAG (Glu)
TGGT1_264420		82	T	A	SNP	23ATA (Ile) /AAA(Lys)
TGGT1_287500	Putative T complex chaperonin	60766	A	C	SNP	772 CTC (Leu) /CGC (Arg)
TGGT1_411100	Phenylalanine-4-hydroxylase	60766	A	C	SNP	772CTC (Leu)/CGC (Arg)
TGGT1_217961	Hypothetical protein	2611	G	T	SNP	110CTC (Leu)/ ATC (Ile)
TGGT1_318880	Hypothetical protein	1122991	G	C	SNP	668GAG (Glu)/ CAG (Gln)
TGGT1_226950	IgA-specific metalloendopeptidase	2826903	G	A	SNP	651 CGA (Arg) / TGA (stop) [650/2646]
