# Supplementary material for: Ablation of an Ovarian Tumor Family Deubiquitinase Exposes the Underlying Regulation Governing the Plasticity of Cell Cycle Progression in Toxoplasma gondii
Source: mBio. 2017 Nov 21;8(6):e01846-17. doi: 10.1128/mBio.01846-17 (PMC5698556; doi:10.1128/mBio.01846-17)
Supplement: TABLE S1 [file mbo006173593st1.pdf]

Table- S1

List of primers:

**HA- epitope tagging**<br>Fwd: 5’-TACTTCCAATCCAATTTAGCGCCTCGTCTTCCTTCG-3’<br>Rev: 5’-TCCTCCACTTCCAATTTTAGCCCCTCGTTGTGCGCCCGT-3’
**TgOTUD3A-CRISPR KO primer**<br>Fwd: 5’-CAATGCCGCTCTGTCGGAACAAGCTTCGCCAGGCTGTA-3’<br>Rev: 5’-GACTCTCCGCCCGCTCCCCGGAATTCATCCTGCAAGTGCA-3’
**TgOTUD3A-CRISPR complementation primer**<br>CRISPR01 Fwd: 5’-GTTCATTGTCGTTTTAGAGCTAGAAATAGCAAG-3’<br>CRISPR01 Rev: 5’-TGAGGTGTGCAACTTGACATCCCCATTTAC -3‘<br>CRISPR02 Fwd: 5'-TCTTCTGAGAGTTTTAGAGCTAGAAATAGCAAG-3‘<br>CRISPR02 Rev: 5'-CTATGGTAACAACTTGACATCCCCATTTAC -3'
**DHFR Cassette with TgOTUD3A –CRISPR site flanking seq**<br>Fwd: 5’-CAATGCCGCTCTGTCGGAACAAGCTTCGCCAGGCTGTA-3’<br>Rev: 5’-GACTCTCCGCCCGCTCCCCGGAATTCATCCTGCAAGTGCA-3’
**TgOTUD3A-CRISPR (DHFR integration check primer)**<br>Fwd1: 5’- CACAGAGTGGACCGAAGGA-3’<br>Rev1: 5’- TGCAACTGTCTCAGGCATTG-3’<br>Rev2: 5’- ACGAATCCAGATGGAGATGG-3’
**TgOTUD3A-CRISPR (KO gDNA sequencing primer)**<br>Seq Pr: 5’-CACAGAGTGGACCGAAGGA-3’
**Competition Assay primer**<br>Wt-HA Fwd: 5’-GATACGAACTGGTATCTGCGATAC-3’<br>Wt-HA Rev: 5’-CCTAGGGAATTCCCGTCCT-3’<br>OTUD3A KO Fwd: 5’-CCGTCACCTCAGAATTCATCC-3’<br>OTUD3A KO Rev: 5’-TTTCACACAGTCTCACCTCGC-3’
**Real-time (TgOTU expression) analysis primer**<br>TgSAG1qrtFwd: 5’-ACACGGCAGGCATCAAAC-3’<br>TgSAG1qrtRev: 5’-ACAACTTGACAGGACCAAGAGTG-3’<br>TgOTUD5qrtFwd: 5'-CACTGCTGAATGGGGAGG-3'<br>TgOTUD5qrtRev: 5'-GGCACCACTGAGTTGTAGTGC-3'<br>TgOTUD1BqrtFwd: 5'-AGGTTATCCCTTGGCAGAAGA-3'<br>TgOTUD1BqrtRev: 5'-GGTATGATAACCTGGACTGCATG-3'<br>TgOTU7qrtFwd: 5'-TTGCTATGAGCCATGCACTC-3'<br>TgOTU7qrtRev: 5'-CTGGAAACCCCAAACAAGC-3'<br>TgOTUD1CqrtFwd: 5'-GAAGACTTCTCTGGAGACGAATG-3'<br>TgOTUD1CqrtRev: 5'-TCTGGGCAGTTGACTCAGAG-3'<br>TgOTU8qrtFwd: 5'-GGCTTGGTTCTACACCGGT-3'<br>TgOTU8qrtRev: 5'-TCGGAGAATGTATCTCCCAAG-3'<br>TgOTUBqrtFwd: 5’-CGACTTCGCTGAAGAAACTGT-3’<br>TgOTUBqrtRev: 5’-TCCTCCACCGTTGCGTAC-3’
